# Methylation of PhoP by CheR Regulates *Salmonella* Virulence

**DOI:** 10.1128/mBio.02099-21

**Published:** 2021-09-21

**Authors:** Yang Su, Jianhui Li, Wenting Zhang, Jinjing Ni, Rui Huang, Zuoqiang Wang, Sen Cheng, Yue Wang, Zhixin Tian, Qiuxiang Zhou, Donghai Lin, Wenjuan Wu, Christoph M. Tang, Xiaoyun Liu, Jie Lu, Yu-Feng Yao

**Affiliations:** a Laboratory of Bacterial Pathogenesis, Department of Microbiology and Immunology, Shanghai Jiao Tong University School of Medicine, Shanghai, China; b Department of Microbiology, School of Basic Medical Sciences, Peking University Health Science Center, Beijing, China; c School of Chemical Science & Engineering, Shanghai Key Laboratory of Chemical Assessment and Sustainability, Tongji University, Shanghai, China; d College of Chemistry and Chemical Engineering, Xiamen University, Xiamen, China; e Department of Laboratory Medicine, Shanghai East Hospital, Tongji University School of Medicine, Shanghai, China; f Sir William Dunn School of Pathology, University of Oxford, Oxford, UK; g Department of Infectious Diseases, Ruijin Hospital, Shanghai Jiao Tong University School of Medicine, Shanghai, China; h School of Life Sciences and Biotechnology, Shanghai Jiao Tong University, Shanghai, China; i Shanghai Key Laboratory of Emergency Prevention, Diagnosis and Treatment of Respiratory Infectious Diseases, Shanghai, China; School of Medicine, Oregon Health & Science University

**Keywords:** methylation, PhoP, methyltransferase, CheR, virulence

## Abstract

The two-component system PhoP/PhoQ is essential for Salmonella enterica serovar Typhimurium virulence. Here, we report that PhoP is methylated extensively. Two consecutive glutamate (E) and aspartate (D)/E residues, i.e., E8/D9 and E107/E108, and arginine (R) 112 can be methylated. Individual mutation of these above-mentioned residues caused impaired phosphorylation and dimerization or DNA-binding ability of PhoP to a different extent and led to attenuated bacterial virulence. With the help of specific antibodies recognizing methylated E8 and monomethylated R112, we found that the methylation levels of E8 or R112 decreased dramatically when bacteria encountered low magnesium, acidic pH, or phagocytosis by macrophages, under which PhoP can be activated. Furthermore, CheR, a bacterial chemotaxis methyltransferase, was identified to methylate R112. Overexpression of *cheR* decreased PhoP activity but increased PhoP stability. Together, the current study reveals that methylation plays an important role in regulating PhoP activities in response to environmental cues and, consequently, modulates Salmonella virulence.

## INTRODUCTION

Salmonella enterica serovar Typhimurium expresses a variety of virulence factors to cause a wide range of diseases in different hosts, from mild enterocolitis to systemic, lethal infection (1–3). *S.* Typhimurium uses diverse regulatory circuits such as two-component signal transduction systems to promote its capacity to disrupt the primary barriers within hosts and evade immune defense. The PhoP/PhoQ system is comprised of the kinase sensor PhoQ and the response regulator PhoP. This two-component system controls various cellular functions by responding to environmental alterations (4–6). PhoQ resides in the bacterial plasma membrane and is activated upon sensing environmental signals such as low concentrations of Mg^2+^ or an acidic pH produced by macrophages. Activation leads to the autophosphorylation of PhoQ, which is followed by the phosphorylation of PhoP (7, 8). PhoP then binds to its target gene promoters with high affinity, stimulating an array of gene transcription (9, 10).

Posttranslational modifications (PTMs), comprising hundreds of potential covalent modifications, are essential in both prokaryotes and eukaryotes to increase protein versatility. PTMs can change protein size, charge, stability, or structure, leading to alteration of protein activity, localization, or interaction with other molecules. In addition, cross talk between different types of PTMs further increases the complexity of protein language. PTMs have been found to be involved in the regulation of gene expression, signal transduction, and bacterial virulence (11–16). The activity of PhoP is governed by a complex network of PTMs, including phosphorylation and acetylation (16, 17). In our previous work, we demonstrated that acetylation of PhoP K201 by the protein acetyltransferase Pat played an important role in Salmonella virulence by regulating the DNA-binding ability of PhoP (17). Recently, we showed that acetyl phosphate-dependent acetylation of PhoP K102 reduced the virulence of *S.* Typhimurium by inhibiting the phosphorylation of PhoP (18). These results highlight the complexity of PhoP PTMs and their important roles in bacterial adaptation to environmental conditions.

Protein methylation catalyzed by *S*-adenosyl-l-methionine (SAM)-dependent methyltransferase is a prevalent PTM and has been shown to affect a number of eukaryotic processes, including protein transport, transcription, protein-protein interactions, and cell signaling (19–21). To date, methylation has been identified on the side chains of many amino acid residues, including lysine, arginine, glutamate, glutamine, methionine, aspartate, and histidine (22–24). Lysine and arginine are the most commonly methylated residues. Lysine residue can be mono-, di-, or trimethylated, while arginine residue can be mono-, asymmetrically, or symmetrically dimethylated (25). Reversible covalent methylation of lysine and arginine residues may increase the signaling potential of substrate proteins and result in multiple physiological consequences (26–29). Besides arginine and lysine methylation, aspartate and glutamate methylation is also abundant, potentially occurring in about 2% of proteins in eukaryotic cells (30). Aspartate and glutamate methylation is similar to dephosphorylation in terms of the charge change which can influence the substrate amino acid residue in three major ways, neutralizing the negative charge, extending the size, and increasing hydrophobicity (30).

Many studies have reported that bacterial protein methylation is widespread and required for virulence in some pathogenic bacteria (31–33). In *S.* Typhimurium, the methyltransferase CheR modifies a series of membrane chemoreceptors during chemotaxis. Afterward, the receptor generates a signal that is transduced via several proteins, which eventually modulates flagellar rotation and results in the movement of the cell (34, 35).

In the present study, we demonstrate that the crucial virulence regulator PhoP can be methylated at multiple residues. Methylation of PhoP inhibits its activity and consequently decreases the virulence of *S.* Typhimurium through different mechanisms, including reducing the phosphorylation of PhoP and impairing PhoP dimerization and DNA-binding abilities. Additionally, we show that PhoP R112 can be methylated by CheR both *in vivo* and *in vitro*. Overexpression of *cheR* can increase PhoP stability while decreasing the survival of *S.* Typhimurium in acidic environments. This is the first report showing PhoP can be methylated and uncovering the significance of PhoP methylation on regulating Salmonella virulence. Importantly, our data show that methylation of PhoP is catalyzed by CheR, a key factor in bacterial chemotaxis. Therefore, these findings provide new perspectives for understanding the underlying mechanisms of how bacteria coordinately regulate chemotaxis and virulence.

## RESULTS

### E8, D9, E107, E108, and R112 can be methylated and are crucial for PhoP activity.

PTMs are employed by both prokaryotes and eukaryotes to diversify and optimize their protein functions. Our previous data showed that PhoP could be acetylated, and this PTM plays an important role in regulating Salmonella virulence (17, 18). Interestingly, mass spectrometry analyses of PhoP revealed many methylation events on PhoP, including E8, D9, E107, E108, and R112 (Fig. S1A to C in the supplemental material). The methylation of E8, E107, E108, and R112 was detected in both log and stationary phases, while the methylation of D9 was only detected in the stationary phase.

10.1128/mBio.02099-21.1 FIG S1 Liquid chromatography-tandem mass spectrometry (LC-MS/MS) of PhoP modified with methylation on E8, D9, E107, E108, and R112. (A to C) 6×His-tagged PhoP was purified from log- or stationary-phase *S*. Typhimurium *phoP* (eWT)-His strain cultured in LB broth and analyzed with LC-MS/MS after trypsin digestion. The methylation of E8, E107, E108, and R112 (A and B) was detected in both log and stationary phases. The methylation of D9 (C) only was detected in stationary-phase. (D) 6×His-tagged PhoP was purified from *S*. Typhimurium *phoP* (eWT)-His strain cultured in M9CA supplemented with 10 mM Mg^2+^ followed by LC-MS/MS after trypsin digestion. Mass spectrometry analysis confirmed that PhoP R112 could be dimethylated at high Mg^2+^. Download FIG S1, TIF file, 2.0 MB. Copyright © 2021 Su et al. 2021 Su et al. https://creativecommons.org/licenses/by/4.0/ This content is distributed under the terms of the Creative Commons Attribution 4.0 International license.

**FIG 1:**
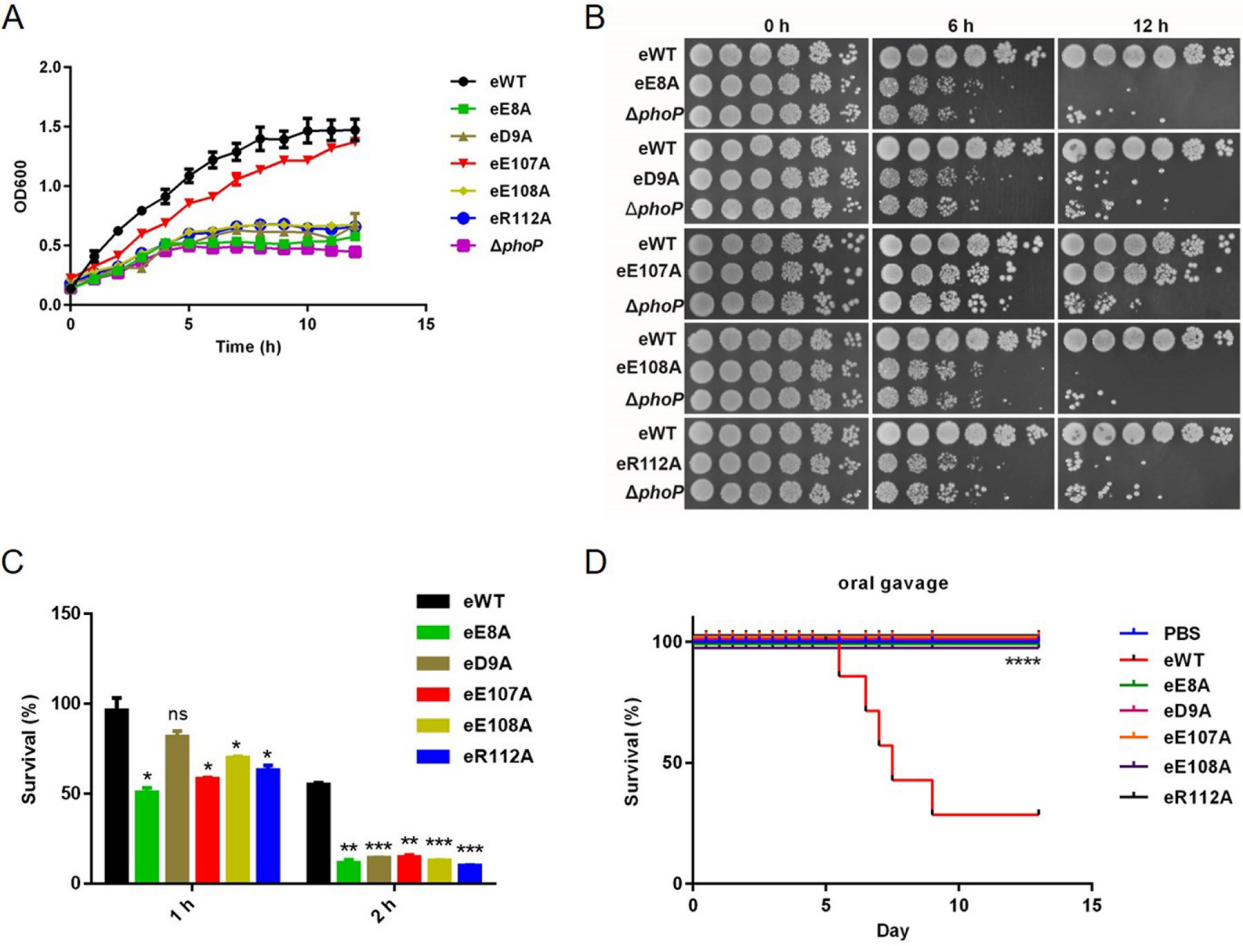
E8, D9, E107, E108, and R112 are important for the activation of PhoP. (A) Growth curves for eWT, eE8A, eD9A, eE107A, eE108A, eR112A, and Δ*phoP* strains. The overnight cultures were diluted to an OD_600_ of 0.1 in M9CA medium supplemented with 8 μM Mg^2+^ and cultured with shaking at 37°C, and OD_600_ was measured each hour. (B) Spot plating assay of eWT, eE8A, eD9A, eE107A, eE108A, and eR112A strains. The overnight cultures grown in LB medium were diluted to an OD_600_ of 0.1 in M9CA medium with a low concentration (8 μM) of Mg^2+^ and continued to growth. At the indicated time points, all samples were collected and normalized to an OD_600_ of 0.008, and then an aliquot of each culture was serially 5-fold diluted from left to right and spotted on LB plates. Plates were photographed after 14 h of incubation at 37°C. (C) Log-phase ATR of eWT and *phoP* mutant strains. Log-phase bacteria were adapted in EG medium at pH 5.8 for 1 h, and then cells were harvested and resuspended in the same volume of EG medium at pH 3.3. After 1 h or 2 h of incubation, viable counts were determined by CFU counting. Survival rate is calculated as the ratio of the number of colonies obtained after and before acid treatment. Data are the averages from three independent experiments. Results are shown as mean ± SD. Statistical difference was calculated between eWT and individual mutant. ***, *P < *0.001; **, *P < *0.01; *, *P < *0.05 by Student's *t* test. (D) Survival rates of mice infected by oral gavage. BALB/c mice were infected by 1.5 × 10^7^ bacteria (wild type or *phoP* mutants) in 200 μl of PBS or an equal volume of PBS as control through oral gavage (seven mice/group). The number of live mice was counted twice per day. Mantel-Cox test was performed between eWT-infected and other mutant Salmonella-infected mice. ****, *P*<0.0001.

To study the physiological relevance of these methyl acceptor site variants to PhoP activity, we first introduced an alanine substitution to each residue individually in PhoP pre-engineered with a C-terminal His tag in chromosome. Because PhoP-PhoQ regulon is involved in *S.* Typhimurium response to environmental stresses, including low concentrations of Mg^2+^ (36), we decided to investigate whether these potential methyl acceptor sites are critical for bacterial response to environmental challenges. We explored the growth and viability of engineered wild-type (eWT), eE8A, eD9A, eE107A, eE108A, and eR112A strains subjected to low concentrations of Mg^2+^ since these strains showed no growth defects in LB broth and M9CA medium (M9 medium broth powder, 11.3 g/L; casamino acids, 1 g/L; glycerol, 2.8 ml/L) supplemented with high concentrations of Mg^2+^(10 mM) (Fig. S2). We found that all *phoP* mutants except eE107A were significantly more sensitive to low concentrations of Mg^2+^ than eWT (Fig. 1A and B). The growth and cell viability of eE8A, eD9A, eE108A, and eR112A dramatically decreased.

10.1128/mBio.02099-21.2 FIG S2 Growth curves of eWT and *phoP* mutants in LB and high-magnesium medium. The overnight cultures of eWT, eE8A, eD9A, eE107A, eE108A, and eR112A strains were diluted to an OD_600_ of 0.01 in fresh LB medium (A) or M9CA medium supplemented with 10 mM Mg^2+^ (B). Cultures were grown at 37°C with shaking, and OD_600_ was measured each hour. Download FIG S2, TIF file, 0.5 MB. Copyright © 2021 Su et al. 2021 Su et al. https://creativecommons.org/licenses/by/4.0/ This content is distributed under the terms of the Creative Commons Attribution 4.0 International license.

As an enteric pathogen, *S.* Typhimurium must endure the acidic environment of the host stomach and phagosome before it can cause systemic infection (37–39). PhoP activation is crucial for the acid tolerance response (ATR), which protects bacteria from severe acid stress (40). Thus, we hypothesized that these mutants could be more vulnerable to acid stress. Indeed, after adaption to mild acid (pH 5.8) for 1 h and then 2 h of incubation with lethal acid (pH 3.3), the survival rate of eWT was higher than 50%, while the survival rates of *phoP* mutant strains were less than 20% (Fig. 1C).

We then infected HeLa cells and the mouse macrophage-like RAW 264.7 cells with eWT or *phoP* mutants to determine bacterial cell invasion and intracellular survival/proliferation, respectively. The invasion levels of the *phoP* mutants were 5- to 9-fold higher than that of eWT (Fig. S3A). Similarly, eWT proliferated by approximately 33-fold from 2 h to 24 h postinfection in macrophages, while all *phoP* mutant strains exhibited only around 10-fold replication in macrophage cells (Fig. S3B). Since activated PhoP can suppress invasion but promote intracellular survival (41), these results suggested that E8, D9, E107, E108, and R112 residues were essential for PhoP activity.

10.1128/mBio.02099-21.3 FIG S3 E8, D9, E107, E108, and R112 are important for the activation of PhoP and contribute to *S*. Typhimurium virulence in mouse model. (A) HeLa cell invasion efficiency by the wild-type strain and *phoP* mutants. HeLa cells were infected at an MOI of 100 by exponential-phase bacterial cultures. At the same time, the number of total alive bacteria was determined by plating an aliquot of culture on LB plates. At 2 h postinfection, cells were lysed, and released intracellular bacteria were enumerated on LB agar plates. Invasion efficiency was calculated by dividing the number of intracellular bacteria with the input alive bacteria and expressed as a percentage. Results are shown as mean ± SD. ***, *P < *0.001; **, *P < *0.01 by Student’s *t* test. Statistical difference was calculated between eWT and individual *phoP* mutant. (B) The proliferation of the wild-type strain and *phoP* mutant strains in macrophages. At 2 h or 24 h postinfection, cells were lysed and plated on LB agar plates, and bacterial colonies were counted. Bacterial replication folds between 2 h and 24 h were calculated. Results are shown as mean ± SD; *, *P < *0.05 by Student’s *t* test. Statistical difference was calculated between eWT and individual *phoP* mutant. (C) Survival rates of mice infected by intraperitoneal injection. BALB/c mice were injected intraperitoneally by 1.5 × 10^5^ bacteria (wild type or *phoP* mutants) in 100 μl PBS or PBS of equal volume as control (seven mice/group). The mortality of mice was recorded twice per day. Mantel-Cox test was performed between eWT-infected and individual *phoP* mutant-infected mice, ****, *P*<0.0001. (D and E) Bacterial burdens in liver and spleen. The livers and spleens were harvested 48 h after oral gavage infection. The number of bacteria was counted. Results are shown as mean ± SD. ***, *P < *0.001 by Student’s *t* test. Statistical difference was calculated between eWT and individual *phoP* mutant. (F) Bacterial burdens in ceca of mice. The ceca from streptomycin-pretreated mice were harvested 48 h after oral gavage infection and prepared as paraformaldehyde-fixed paraffin section. These sections were stained for lipopolysaccharide (LPS) (red), actin (green), and nuclei with DAPI (4′,6-diamidino-2-phenylindole; blue). Images are pseudocolor representations at ×200 magnification. (G) H&E-stained ceca of mice. The ceca of the wild type- or *phoP* mutant-infected mice were fixed and embedded in paraffin, and then 5-μm-thin sections were cut and stained with H&E. (H) Neutrophil infiltration in ceca. The paraffin section was stained by hematoxylin and incubated with the anti-MPO antibody and followed by immunohistochemistry. Blue indicates the nucleus, and claybank indicates polymorphonuclear neutrophils (PMN). Download FIG S3, TIF file, 1.7 MB. Copyright © 2021 Su et al. 2021 Su et al. https://creativecommons.org/licenses/by/4.0/ This content is distributed under the terms of the Creative Commons Attribution 4.0 International license.

To further determine the significance of the residues E8, D9, E107, E108, and R112 of PhoP to *S.* Typhimurium virulence, we employed a streptomycin-pretreated mouse model of oral gavage infection. After infection, the mice were monitored over a 15-day period. As shown in Fig. 1D, there were no deaths in the *phoP* mutant strains-infected mice and the phosphate-buffered saline (PBS) control group throughout the whole experimental period. In contrast, mice infected with eWT began dying on day 5.5 postinfection, and only 2 eWT-infected mice survived the infection. Quantification of the abundance of eWT and *phoP* mutant strains in spleen and liver of mice on day 2 postinfection demonstrated that recovery of *phoP* mutant strains was significantly less than that of eWT in both tissues (Fig. S3C and Fig. S2D). Intraperitoneal injection resulted in similar results as oral administration (Fig. S3E).

We further examined bacterial colonization and the histopathologic features affected by cecal inflammation. Immunohistochemistry showed that eWT colonized in ceca of streptomycin-pretreated mice. In contrast, *phoP* mutant strains were barely detected in ceca (Fig. S3F). Morphology analyses of hematoxylin and eosin (H&E)-stained colons confirmed that desquamation in the epithelial layer was obvious in eWT-infected mice, while virtually no desquamation was observed in mice infected by *phoP* mutant strains (Fig. S3G). Polymorphonuclear neutrophils (PMNs) are the most abundant effector leukocytes in the innate immune system. We observed the PMN infiltration of the submucosa, the lamina propria, and the epithelial layer and the transmigration of PMN into the intestinal lumen in eWT-infected mice (Fig. S3H). However, no PMNs were observed in eE8A- and eD9A-infected mice, and only a few PMNs were observed in e107A-, eE108A-, and eR112A-infected mice.

### The roles of E8, D9, E107, E108, and R112 in PhoP activation.

**FIG 2:**
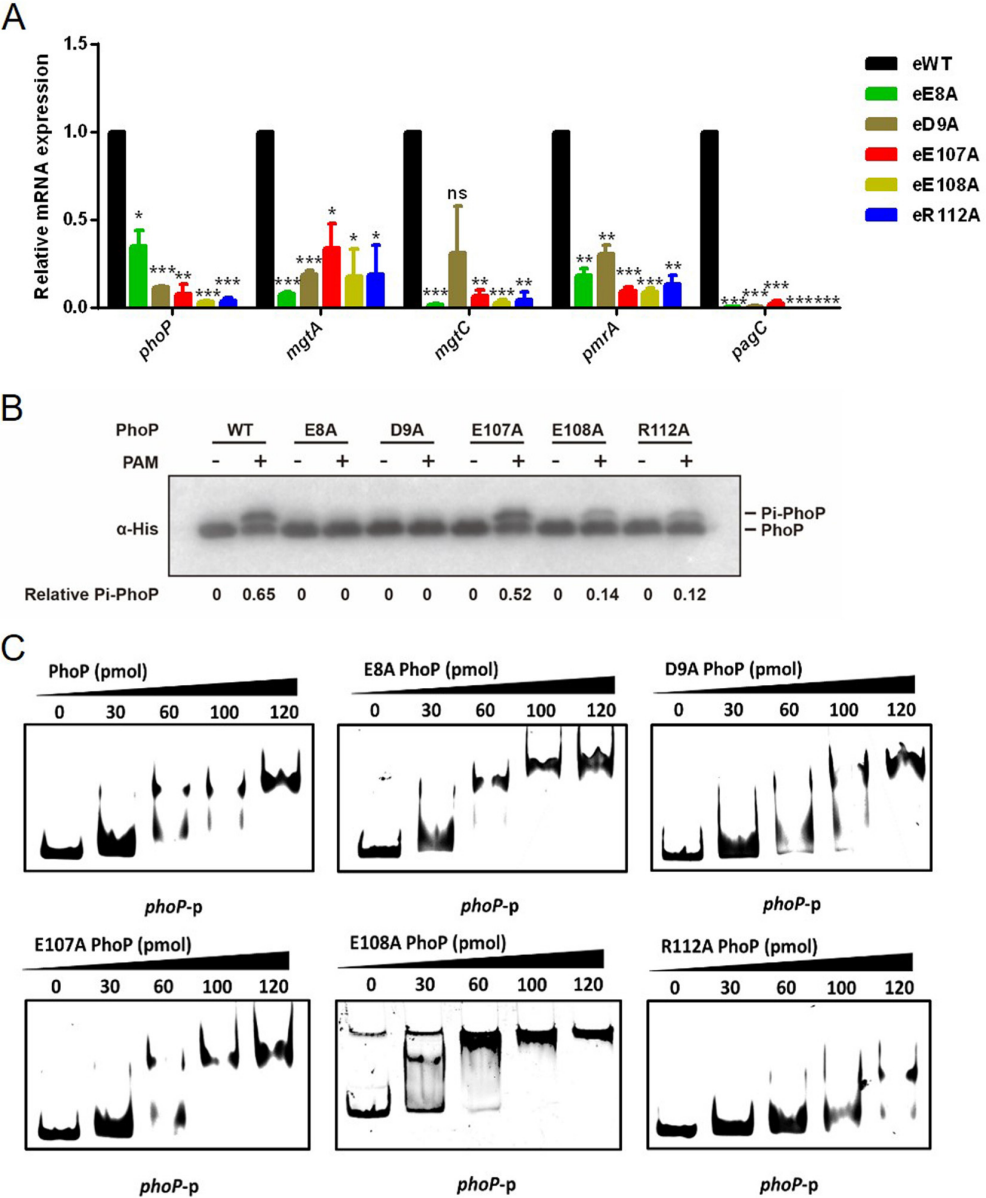
The influence of E8, D9, E107, E108, and R112 mutation on PhoP activity. (A) Reverse transcription-quantitative PCR (qRT-PCR) analysis of *phoP* and PhoP target genes in eWT, eE8A, eD9A, eE107A, eE108A, and eR112A cells. Total RNA was isolated from eWT, eE8A, eD9A, eE107A, eE108A, and eR112A cells grown in M9CA medium with low concentration of Mg^2+^. All mRNA levels were normalized to the control 16S rRNA mRNA level and expressed as the fold change in mRNA levels over eWT. Data are shown as mean ± SD with three biological replicates. Statistical difference was calculated between eWT and individual *phoP* mutant. ns, no significance; ***, *P < *0.001; **, *P < *0.01; *, *P < *0.05 by Student's *t* test. (B) Phosphorylation of PhoP variants. The purified proteins were incubated with 20 mM PAM for 1 h and resolved by Phos tag gel and detected by Western blotting with anti-His antibody. The upper band is the phosphorylated PhoP (Pi-PhoP), and the lower band is the unphosphorylated PhoP. Experiments were performed in triplicates, and representative results are shown. (C) DNA-binding ability of PhoP variants by EMSA. The indicated amounts of PhoP and variants were incubated with the *phoP* promoter, and then the mixtures were analyzed by EMSA.

The above-mentioned five amino acid residues are located in the N-terminal receiver domain of PhoP and are highly conserved across different bacterial species (Fig. S4A). Our existing data showed that these residues were crucial for PhoP function, so we speculated that they would be involved in regulating the transcriptional activity of PhoP. Therefore, we quantified the transcription level of PhoP itself and its target genes, including *mgtA*, *mgtC*, *pmrA*, and *pagC*, in these mutants. The quantitative real-time PCR (qPCR) results showed that, compared with the eWT, eE8A, eD9A, eE107A, eE108A, and eR112A had lower transcription levels of *phoP* itself and PhoP target genes (Fig. 2A).

10.1128/mBio.02099-21.4 FIG S4 Structure analysis and dimer formation of PhoP E8, D9, E107, E108, and R112. (A) Conservation analysis of PhoP E8, D9, E107, E108, and R112. Asterisks denote the conserved E8, D9, E107, E108, and R112. The sequences were analyzed by BioEdit 7.0. (B) Interactions between E8, D9, E107, E108, and R112 and other residues. (B, Left) E8 and D9 are involved in forming salt bridges with K102, which might regulate PhoP phosphorylation via acetylation. (B, Right) E107, E108, and R112 are located within α4-β5-α5 motif, which might regulate PhoP dimerization. The relative distance between R112 and DNA is closer than the other residues, indicating its higher binding affinity with the *phoP* promoter. (C) Phosphorylation of PhoP. PhoP was incubated with 20 mM PAM for different time as indicated. The samples were resolved on 10% SDS-PAGE gel containing Phos tag followed by Western blotting using anti-His antibody. (D) Dimer formation of PhoP variants. PhoP and variants were subjected to cross-linking with 1 mM DSS. The samples were analyzed by Western blotting using anti-His antibody. Download FIG S4, TIF file, 1.9 MB. Copyright © 2021 Su et al. 2021 Su et al. https://creativecommons.org/licenses/by/4.0/ This content is distributed under the terms of the Creative Commons Attribution 4.0 International license.

The crystal structures revealed that the side chains of the above-mentioned five amino acid residues interacted with the side chains of other key residues (42, 43). E8 and D9 are involved in the formation of salt bridges with K102, which might regulate PhoP phosphorylation via acetylation (18). E107, E108, and R112 are located within α4-β5-α5 motif, which might regulate PhoP dimerization (Fig. S4B). Therefore, modifications of these residues’ side chains might influence PhoP protein structure and, consequently, change PhoP activities. To examine this, we tested whether the above five variants of PhoP possessed altered phosphorylation levels. Since acetyl phosphate has been shown to serve as both acetyl and phosphoryl donor for PhoP, and acetylation and phosphorylation have opposite effects on PhoP (18), we decided to use phosphoramidate (PAM), which can only serve as phosphodonor, to observe the effect of individual mutation on PhoP phosphorylation without interference of acetylation (44). PhoP and its variants were overexpressed and purified by Ni-nitrilotriacetic acid (Ni-NTA) agarose resin to homogeneity. We then synthesized PAM to perform *in vitro* phosphorylation assay followed by Western blotting. The result showed that the amount of the phosphorylated PhoP (Pi-PhoP) increased over time with incubation of 20 mM PAM, suggesting our phosphorylation system works well (Fig. S4C). The E8A and D9A variants cannot be phosphorylated, while the phosphorylation of E107A was comparable to that of the wild-type protein. Interestingly, the phosphorylation of E108A and R112A dramatically decreased (Fig. 2B).

Besides phosphorylation, dimerization is also a prerequisite for PhoP transcription (45, 46). We examined the dimer formation ability of PhoP variants using disuccinimidyl suberate (DSS) cross-linking. As expected, E8A, D9A, and E107A showed less dimerization than the wild-type PhoP, while E108A and R112A exhibited dramatically impaired dimerization (Fig. S4D).

PhoP-mediated transcription relies on its binding to PhoP boxes within the promoters of target genes (9, 47). To test DNA-binding ability of E8A, D9A, E107A, E108A, and R112A variants, electrophoretic mobility shift assays (EMSA) were carried out by incubating these purified proteins with the *phoP* promoter. Results showed that the PhoP R112A variant had lower binding affinity with the *phoP* promoter than the other five proteins (Fig. 2C).

### E8 and R112 methylation decreases under PhoP/PhoQ-activating conditions.

So far, we have demonstrated the importance of E8, D9, E107, E108, and R112 residues for PhoP function. Next, we want to investigate whether the methylation on these residues is of physiological relevance. To study the role of PhoP methylation *in vivo*, we tried to generate antibodies against peptides containing methylated E8, D9, E107, E108, or R112. Unfortunately, methylation antibodies of D9, E107, and E108 PhoP were unsuccessful due to the hydrophobicity of peptides. We only succeeded in raising antibodies specifically recognizing E8 or mono-R112 methylated PhoP (here referred to as α-PhoP E8me or α-PhoP R112me1). Western blot analysis showed that α-PhoP E8me specifically recognized methylated PhoP peptides at E8 and did not cross-react with unmodified PhoP peptides. The α-PhoP R112me1 distinguished methylated PhoP at R112 and the R112A variant as well (Fig. S5).

10.1128/mBio.02099-21.5 FIG S5 Specificity of α-PhoP E8me and α-PhoP R112me1 antibody. (A) Specificity of α-PhoP E8me polyclonal antibody was validated by incubation with methylated peptide and unmethylated peptide. (B) Specificity of α-PhoP R112me1 polyclonal antibody was validated by incubation with PhoP WT and PhoP R112A proteins at different dilutions of antibody. An arginine mutation to alanine will result in the loss of the side chain and failure to be recognized by α-PhoP R112me1 antibody. Download FIG S5, TIF file, 0.1 MB. Copyright © 2021 Su et al. 2021 Su et al. https://creativecommons.org/licenses/by/4.0/ This content is distributed under the terms of the Creative Commons Attribution 4.0 International license.

**FIG 3:**
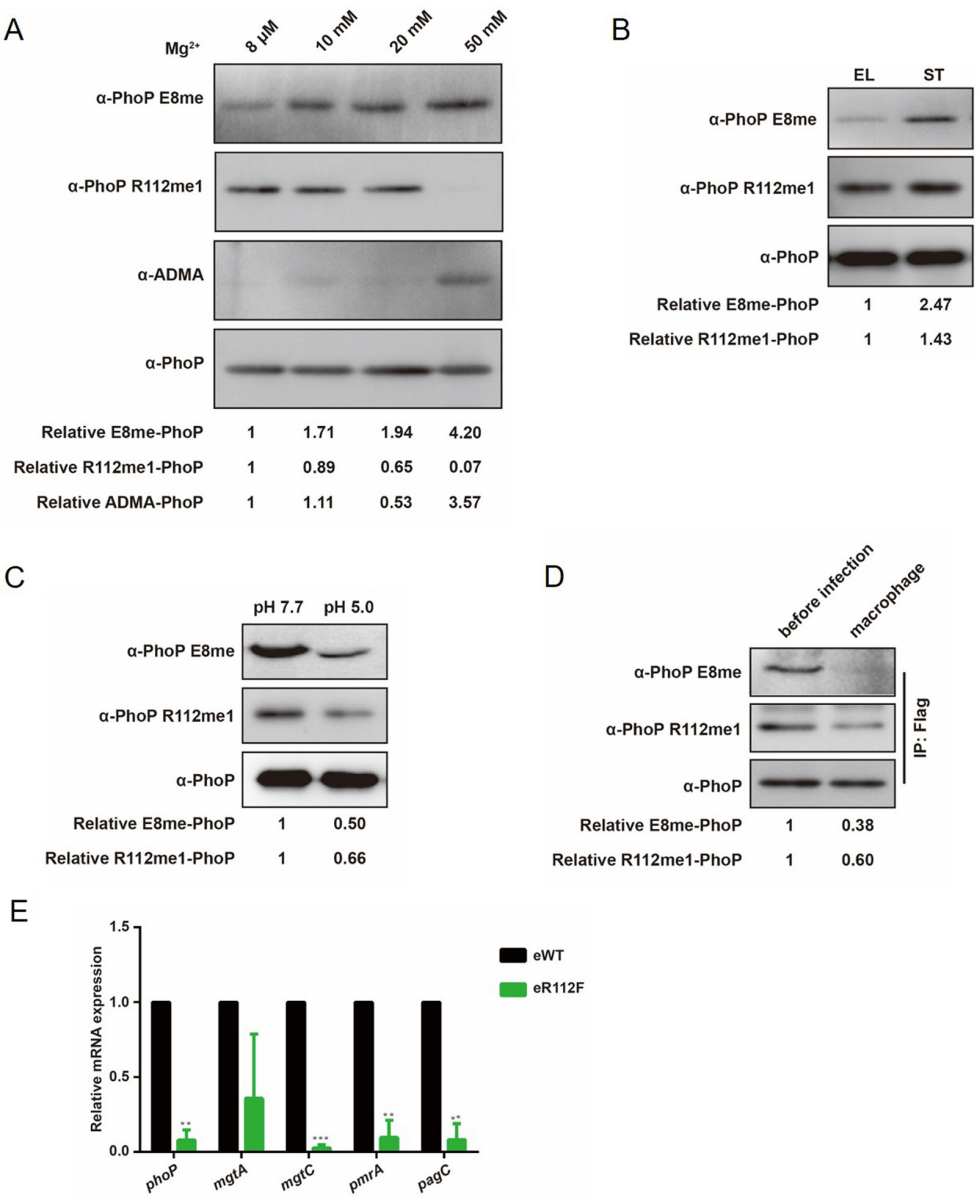
E8 and R112 methylation levels decrease under PhoP/PhoQ-activating conditions. (A) Methylation levels of PhoP under different concentrations of magnesium. The 6×His-tagged PhoP was purified by Ni-NTA column from the log-phase wild-type strain cultured in M9CA medium with different concentrations of magnesium. The methylation levels of the purified PhoP were determined by anti-asymmetric dimethylation antibody (α-ADMA), anti-PhoP E8me, and anti-PhoP R112me1; anti-PhoP antibody was used as a loading control. (B) Methylation levels of PhoP E8 and R112 in log and stationary phases. The 6×His-tagged PhoP was purified from the wild-type strain cultured in LB broth at log or stationary phase. E8 and R112 methylation levels were determined by anti-PhoP E8me or anti-PhoP R112me1, respectively. (C) Methylation level of PhoP after acid stimulation. The wild-type strain was cultured in EG medium to log phase at pH 7.7 and stimulated at pH 5.0 for 1 h. The methylation levels of purified PhoP (His tag) from pH 7.7 and pH 5.0 were determined by anti-PhoP E8me and anti-PhoP R112me1. (D) Methylation level of PhoP in macrophages. After infecting RAW 264.7 cells for 24 h, the intracellular bacteria were harvested for IP assay. The immunoprecipitated PhoP was determined by anti-PhoP E8me and anti-PhoP R112me1. Western blotting was independently repeated at least three times. (E) qRT-PCR analysis of *phoP* and PhoP target genes in eWT and eR112F cells. Results were shown as mean ± SD. Statistical difference was calculated between eWT and eR112F. ***, *P < *0.001; **, *P < *0.01 by Student's *t* test.

In Salmonella, PhoP is an Mg^2+^-responsive transcriptional regulator whose activity decreases as the Mg^2+^ concentration increases (4, 48). To determine whether Mg^2+^ concentration alters the methylation levels of E8 or R112 *in vivo*, His-tagged PhoP proteins were purified from Salmonella grown in M9CA medium supplemented with different concentrations of Mg^2+^ and analyzed by Western blotting. We observed a positive correlation between the methylation levels of endogenous E8 and Mg^2+^ concentrations (Fig. 3A). To our surprise, the monomethylation level of endogenous R112 gradually decreased when Mg^2+^ concentrations increased. Given that arginine *N*-methyltransferases can further catalyze the formation of asymmetric dimethylarginine (ADMA) or symmetric dimethylarginine (SDMA) (49), we hypothesized that most of the monomethylarginine was catalyzed to ADMA at high Mg^2+^ (50 mM). Indeed, the increase of ADMA level of PhoP paralleled that of Mg^2+^ concentration as detected by ADMA antibody (Fig. 3A). Mass spectrometry analysis confirmed that R112 was dimethylated at high Mg^2+^ (Fig. S1D).

The activation of PhoP is also correlated with bacterial growth phase and responsive to environmental pH (4, 6, 50). If methylation of PhoP inhibits the activation of PhoP, we should be able to observe a reduced methylation level of E8 and/or R112 in PhoP-activating conditions, such as log phase or moderate acidic pH (4, 50). Results showed an approximately 147% increase of methylated E8 from log phase to stationary phase (Fig. 3B), while approximately 50% of methylation E8 dropped upon switching bacteria from pH 7.7 to pH 5.0 (Fig. 3C). Although the influence of growth phase and environmental pH on the methylation level of R112 was less, we still found that methylation of R112 was lower in PhoP-activating conditions, including log phase and acidic pH (Fig. 3B and C).

Phagocytosis by macrophages can increase PhoP activity in Salmonella (51). If methylation of E8 and R112 inhibits the activation of PhoP, we speculated that methylation level of E8 and R112 might decrease during bacterial phagocytosis by macrophages. To address this hypothesis, PhoP was immunoprecipitated from intramacrophage bacteria after 24-h infection of RAW 264.7 cells by Salmonella and analyzed by Western blotting by use of α-PhoP E8me or α-PhoP R112me1. As expected, methylation levels of PhoP E8 and R112 in intracellular bacteria decreased dramatically compared with those of bacteria grown in LB medium (Fig. 3D).

Since an arginine-to-phenylalanine (F) mutation could introduce hydrophobicity and mimic constitutively methylated arginine (52), we mutated R112 to phenylalanine to test the relationship between methylation and PhoP activity. As shown in Fig. 3E, the transcription of *phoP* and PhoP target genes in eR112F decreased dramatically, suggesting that methylation might inhibit PhoP activity *in vivo*.

### PhoP is a substrate of CheR.

**FIG 4:**
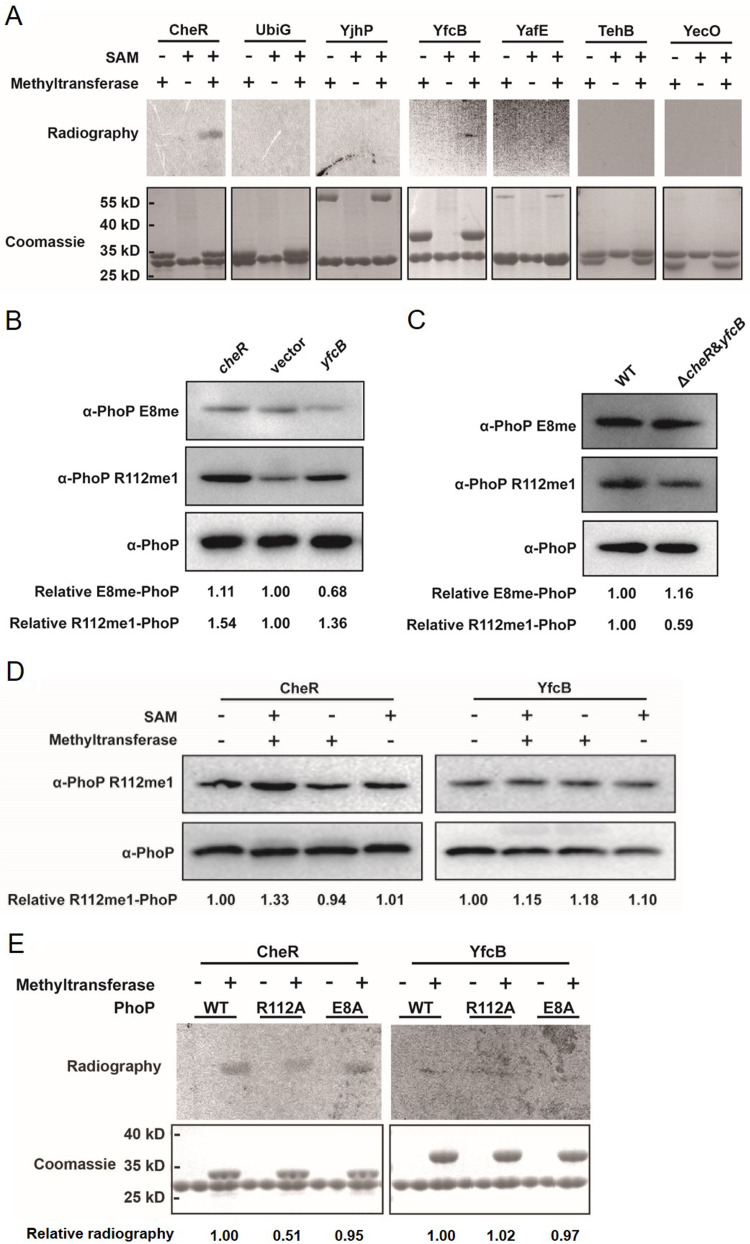
PhoP is a substrate of CheR. (A) Autoradiogram showing methylation activity of indicated methyltransferases using tritiated SAM as a methyl group donor, and PhoP was used as the substrate (autoradiogram, top; Coomassie staining, bottom). (B) Methylation levels of PhoP E8 and R112 in cells with *cheR* or *yfcB* overexpression. (C) Methylation levels of PhoP E8 and R112 in the wild-type strain and *cheR*/*yfcB* double deletion mutant. (D) CheR and YfcB methylate PhoP *in vitro*. Purified His-tagged PhoP was incubated with CheR or YfcB using SAM as a methyl group donor, and then the samples were resolved on SDS-PAGE and detected by Western blotting with anti-PhoP R112me1. (E) Autoradiogram showing methylation activity of CheR, YfcB using tritiated SAM as a methyl group donor, and PhoP, E8A, or R112A were used as the substrates (autoradiogram, top; Coomassie staining, bottom).

In order to identify the methyltransferase(s) acting on PhoP, we analyzed the genomic sequence of *S.* Typhimurium strain 14028S, and 9 potential protein methyltransferase-encoding genes (i.e., *yafE*, *yafS*, *tehB*, *STM14_1982*, *yecO*, *cheR*, *ubiG*, *yjhP*, and *yfcB*) were identified. We then knocked out or overexpressed all these genes individually in the wild-type strain and examined the transcriptional levels of *phoP* and PhoP target genes in the above strains (data not shown). However, the transcriptional levels of *phoP* and PhoP target genes did not change, which means that this strategy cannot identify PhoP methyltransferase. Meanwhile, we successfully purified 7 out of 9 candidate methyltransferase proteins from E. coli, followed by *in vitro* methyltransferase assay using tritiated *S*-adenosylmethionine (^3^H-SAM) as the methyl donor. Autoradiography showed that PhoP could be methylated by CheR and YfcB (Fig. 4A). Since CheR and PhoP have similar mobility on SDS-PAGE, we incubated CheR alone with ^3^H-SAM and found that CheR was not automethylated, indicating CheR transferred methyl groups directly to PhoP (Fig. S6A).

10.1128/mBio.02099-21.6 FIG S6 CheR and PhoP stability. (A) Methylation of PhoP by CheR *in vitro*. Autoradiogram showed methylation activity of CheR was detected when tritiated SAM (as a methyl group donor) and PhoP (as a substrate) coexisted. (B) The pan-methylation levels of PhoP from the wild-type strains and mutants. 6×His-tagged PhoP was purified by Ni-NTA column from the wild type and the other two knockout strains at log phase cultured in LB medium. The pan-methylation levels were determined by anti-pan methylation antibody (α-Pan methyl); the anti-PhoP antibody was used as a loading control. (C) Relative abundance of the methylated R112 identified by LC-MS/MS. PhoP-6×His proteins from WT and Δ*cheR* were purified and digested by trypsin followed by mass spectrometry analysis. The relative abundance of R112-methylated residues was calculated by dividing the average area (representing peptide intensity) of R112-methylated peptides by the average area of total R112-containing peptides. (D) Stability of PhoP R112A. The eWT and eR112A cells were harvested at the indicated time points after treatment with 1 mg/ml chloramphenicol. The amount of PhoP was assessed by anti-His antibody, and α-DnaK antibody was used as a loading control. Download FIG S6, TIF file, 2.3 MB. Copyright © 2021 Su et al. 2021 Su et al. https://creativecommons.org/licenses/by/4.0/ This content is distributed under the terms of the Creative Commons Attribution 4.0 International license.

The methylation level of PhoP was measured by Western blotting in overexpression strain or knockout strain of *cheR* or *yfcB*. Results showed that the methylation level of R112 increased after overexpression of *cheR* or *yfcB*, but the methylation level of E8 did not increase (Fig. 4B). Both individual deletion mutants showed a similar pan-methylation level of PhoP to the parental strain (Fig. S6B), but the methylation level of R112 reduced in the *cheR*/*yfcB* double deletion mutant (Fig. 4C). No obvious difference in the methylation level of E8 was observed between the wild-type strain and double deletion mutant strain (Fig. 4C).

To further clarify which protein is the contributing methyltransferase of PhoP R112, we incubated recombinant CheR or YfcB with PhoP in the presence of SAM. Western blot analysis showed that only CheR could increase the methylation signal of PhoP at R112 (Fig. 4D). Moreover, we performed the same methylation assay in the presence of ^3^H-SAM with the wild-type PhoP, E8A, and R112A variants. A marked decrease in ^3^H-SAM incorporation was observed when the R112A variant was incubated with CheR (Fig. 4E). In contrast, the E8A variant and the wild-type PhoP demonstrated comparable levels of ^3^H-SAM incorporation. Meanwhile, we found the methylation levels of the wild-type PhoP, E8A, and R112A variants were similar when incubated with YfcB.

We further used mass spectrometry to confirm the role of CheR in R112 methylation. PhoP was purified from the *cheR*/*yfcB* double deletion mutant and incubated with CheR or YfcB with or without SAM. The mass spectrometry results showed that all 21 R112-containing peptides from PhoP treated by CheR in the presence of SAM were methylated at R112, while none of 29 R112-containing peptides from PhoP treated by CheR without SAM were methylated at R112. In contrast, methylation of E8, D9, E107, and E108 was not detected in the CheR treatment group. Methylation of E107 was identified in about 10% (6/62) peptides of YfcB treatment group (Data Set S1). To examine the role of *cheR* in PhoP R112 methylation *in vivo*, we analyzed the abundance of methylated R112-containing peptides in WT and Δ*cheR* by mass spectrometry. The results showed that the relative abundance of methylated-R112 containing peptides was reduced by 80% in Δ*cheR* compared to that in WT (Fig. S6C; Data Set S2).

10.1128/mBio.02099-21.9 DATA SET S1 *In vitro* MS data. Purified His-tagged PhoP was incubated with CheR or YfcB using SAM as a methyl group donor, and then the samples were digested by trypsin followed by mass spectrometry analysis. Download Data Set S1, XLSX file, 0.02 MB. Copyright © 2021 Su et al. 2021 Su et al. https://creativecommons.org/licenses/by/4.0/ This content is distributed under the terms of the Creative Commons Attribution 4.0 International license.

10.1128/mBio.02099-21.10 DATA SET S2 *In vivo* MS data. We purified 6×His-tagged PhoP from WT and Δ*cheR S*. Typhimurium 14208 cultured in LB broth, and it was analyzed with LC-MS/MS after trypsin digestion. The relative abundance of total R112 and R112-methylated residues was calculated. Download Data Set S2, XLSX file, 0.02 MB. Copyright © 2021 Su et al. 2021 Su et al. https://creativecommons.org/licenses/by/4.0/ This content is distributed under the terms of the Creative Commons Attribution 4.0 International license.

### CheR stabilizes PhoP in an inactive form by methylation.

**FIG 5:**
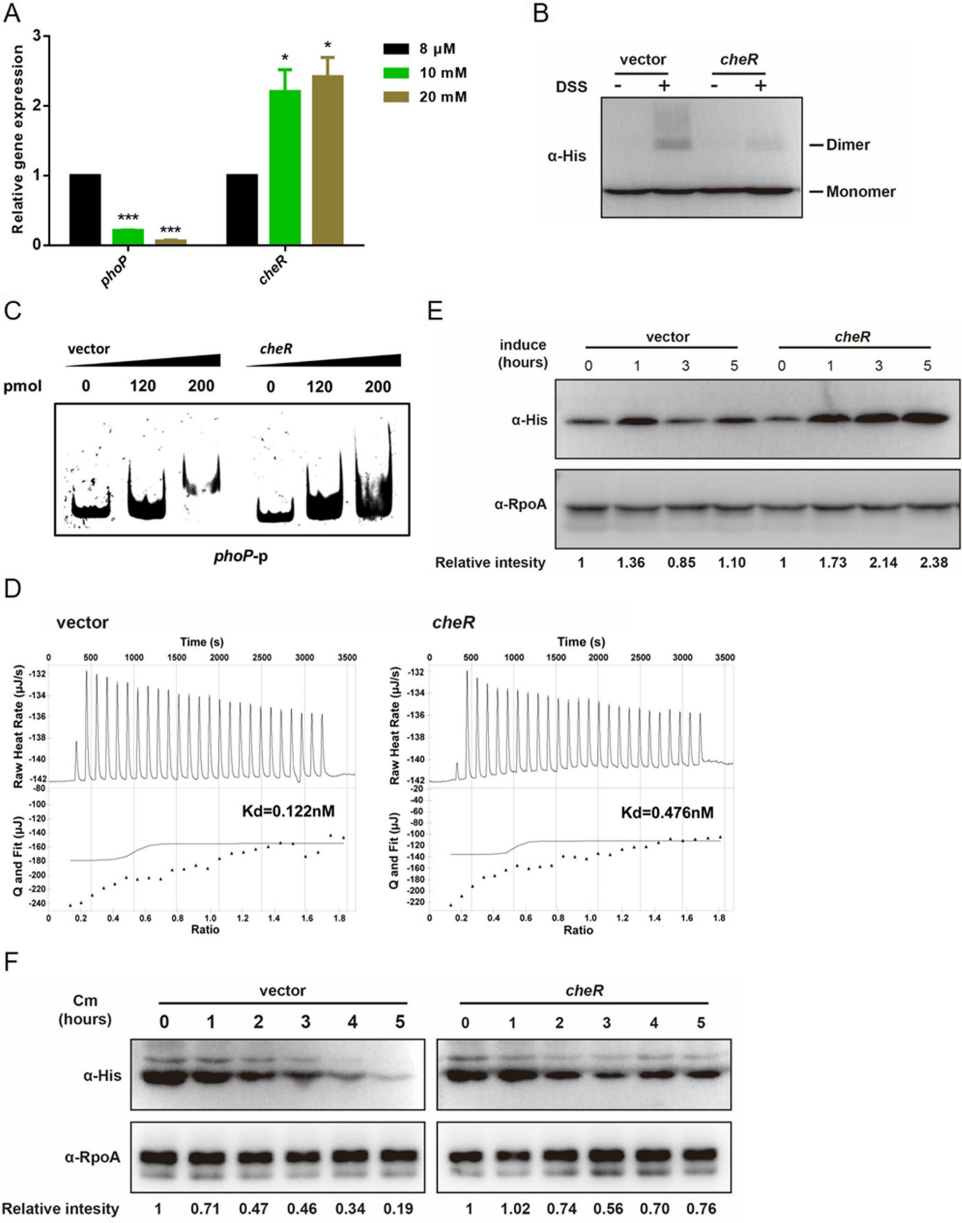
Overexpression of *cheR* decreases PhoP activity but increases PhoP stability. (A) Transcriptional levels of *phoP* and *cheR* under different concentrations of magnesium. Bacteria were harvested from the wild-type cells at log phase, cultured in M9CA medium supplemented with different concentrations of magnesium, and subjected to total RNA isolation. The relative expression level of indicated gene at 8 μM Mg^2+^ was set as 100%. Gene expression levels at higher concentrations of Mg^2+^ (10 mM or 20 mM) were calculated as the fold change to that at 8 μM Mg^2+^, and statistical difference was calculated. Results were shown as mean ± SD. ***, *P < *0.001; **, *P < *0.01 by Student's *t* test. (B) Dimer formation of PhoP with *cheR* overexpression. After overexpression of *cheR* in *S.* Typhimurium, His-tagged PhoP was purified and incubated with DSS, and then samples were analyzed by Western blotting using anti-His antibody. (C) DNA-binding activity of PhoP with *cheR* overexpression by EMSA. The indicated amounts of PhoP purified from *S.* Typhimurium after overexpression of *cheR* were incubated with the *phoP* promoter and analyzed by EMSA. (D) DNA-binding activity of PhoP by ITC. The *phoP* promoter DNA at a concentration of 6.15 μM was titrated into a cell containing 0.6 μM PhoP purified from *cheR*-overexpressing cells or PhoP from control cells at 25°C. DNA was resuspended in buffer to match the PhoP desalting buffer. Data fitting was performed with NanoAnalyze (Malvern). (E) Protein levels of PhoP with *cheR* overexpression. The cells were harvested at the indicated time points after overexpression of *cheR*. The amount of PhoP was assessed by anti-His antibody, and anti-RpoA was used as a loading control. (F) Overexpression of *cheR* increased PhoP stability. After overexpression of *cheR* for 1 h, the cells were treated with 1 mg/ml chloramphenicol and then harvested at the indicated time points. The amount of PhoP was assessed by anti-His antibody, and anti-RpoA was used as a loading control.

Next, we decided to examine the role of CheR in regulating PhoP activities *in vivo*. We determined the transcription levels of *cheR* responding to different Mg^2+^ concentrations, which were shown to be positively correlated with PhoP methylation level (Fig. 3A). Consistent with the kinetics of PhoP methylation, we observed an elevated transcription level of *cheR* along with an increase of Mg^2+^ concentration (Fig. 5A).

Since R112A mutation inhibited the transcriptional activity of PhoP through impairing PhoP DNA-binding ability and dimerization (Fig. 2C and Fig. S4D), we speculated that R112 methylation could have a similar effect. If overexpression of *cheR* impairs the dimerization of PhoP and inhibits the DNA-binding ability of PhoP, we could argue that CheR indeed catalyzes the methylation of R112 and thus inactivates PhoP. As expected, PhoP purified from *cheR*-overexpressing Salmonella cells exhibited both much weaker dimerization (Fig. 5B) and impaired DNA-binding ability (Fig. 5C) compared with PhoP purified from the bacteria with an endogenous level of *cheR* expression. Then, we further used isothermal titration calorimetry (ITC) to examine the real-time binding of PhoP isolated from either the wild-type strain or *cheR*-overexpressing strain to *phoP* promoter. The dissociation constant (*K_d_*) values of PhoP to DNA were 0.122 nM for the former and 0.476 nM for the latter, respectively (Fig. 5D), indicating weaker affinity of methylated PhoP to DNA.

Interestingly, overexpression of *cheR* caused PhoP protein accumulation (Fig. 5E). This phenomenon raised an intriguing possibility that methylation might increase the stability of PhoP. To test this hypothesis, the stability of PhoP was measured by adding chloramphenicol, which inhibited protein synthesis. Western blot analysis results showed the half-life of PhoP substantially increased in *cheR*-overexpressing cells (Fig. 5F). After 5 h treatment, more than 70% PhoP from *cheR*-overexpressing cells still existed, while less than 20% PhoP was left in control cells. Consistent with these findings, R112A showed a shorter half-life than the wild-type PhoP (Fig. S6D), suggesting that the side chain of R112 is crucial for PhoP interaction with protease(s) and R112 methylation might block protease(s) access to PhoP.

### CheR inhibits Salmonella acid resistance and intracellular survival but promotes bacterial invasion.

**FIG 6:**
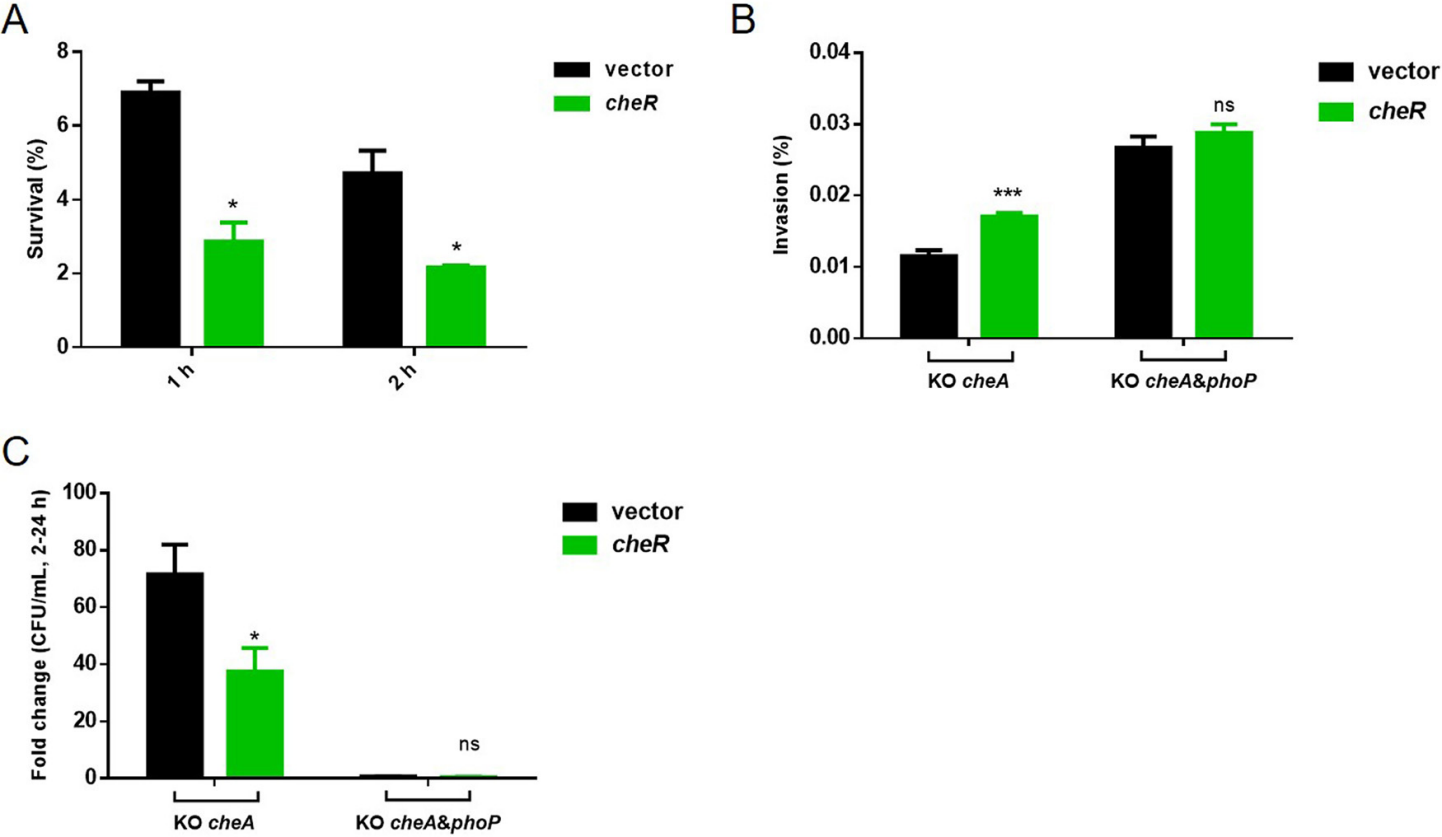
CheR inhibits Salmonella acid resistance and its survival in macrophage but promotes bacterial invasion. (A) Survival of bacteria in acid response after overexpression of *cheR*. Empty vector strain or *cheR*-overexpressing strain was grown for 1 h in EG medium at pH 7.7. Then, the cells were harvested and resuspended in the medium at pH 3.3. After 1 h or 2 h of incubation, viable cells were counted. Survival rate is calculated as the ratio of the number of colonies obtained after and before acid treatment. Results are shown as mean ± SD. Statistical difference was calculated between the vector strain and the *cheR*-overexpressing strain at a given time point. *, *P < *0.05. (B) HeLa cell invasion efficiency of the wild-type and *cheR-*overexpressing strains. HeLa cells were infected with log-phase LB cultures at an MOI of 100. The number of bacteria was determined by plating an aliquot of culture on plates. At 2 h postinfection, cells were lysed and then plated on LB agar plates, and bacterial colonies were counted to calculate invasion efficiency. Results are shown as mean ± SD. Statistical difference was calculated between the vector strain and the *cheR*-overexpressing strain at the indicated genetic background. ns, no significance; ***, *P < *0.001 by Student's *t* test. (C) Proliferation of the wild-type and *cheR-*overexpressing strains in RAW 264.7 macrophage cells. At 2 h or 24 h postinfection, cells were lysed and plated on LB agar plates, and bacterial colonies were counted. Bacterial replication fold between 2 h and 24 h was calculated. Results are shown as mean ± SD; *, *P < *0.05 by Student's *t* test.

When *S.* Typhimurium passes the intestinal tract, it can be internalized by macrophages. Survival and growth in macrophage phagosomes, where the pH is as low as 4 to 5, is crucial for *S.* Typhimurium virulence (53, 54). As a transcriptional activator, PhoP is essential for the replication of *S.* Typhimurium within this acidic intracellular compartment (55). Since the R112A mutation inhibits cell viability when bacteria encounter acid stress, we hypothesized that methylation of PhoP might unfavor intracellular bacterial survival as well. To test this, we measured the survival rate of *cheR*-overexpressing Salmonella cells during acid challenge. We found that overexpression of *cheR* led to about 50% reduction in bacterial survival compared with the control strain (Fig. 6A).

About 60% of bacterial genomes contain gene clusters encoding the signaling proteins for chemosensory pathways that mediate chemotaxis. Among these signaling proteins is CheR, which can methylate bacterial chemotaxis receptors at specific glutamate residues within coiled-coil regions of their cytoplasmic domains (56). Previous studies in other species showed that mutation of *cheR* abolished bacterial chemotaxis (57). Chemotaxis is a key feature that determines host colonization and bacterial virulence (58, 59). Since CheR is a regulator of chemotaxis, we speculate it may be involved in Salmonella invasion and intracellular survival. Therefore, to exclude the chemotaxis alterations caused by *cheR* and dissect the effect of *cheR* as a methyltransferase on related phenotypes, we knocked out the crucial chemotaxis gene *cheA* and blocked the chemotaxis pathway (60, 61). First, the effect of *cheA* deletion on Salmonella chemotaxis was confirmed by swimming assay (data not shown). We then performed invasion and intracellular survival assays in the *cheA* deletion background. As stated previously, activated PhoP can inhibit invasion but promote intracellular survival. Fig. 6B showed that the invasion rate increased by about 50%, owing to *cheR* overexpression. However, when *phoP* was simultaneously deleted in the *cheA* knockout strain, the overexpression of *cheR* did not further promote the invasion, indicating the effect of *cheR* is dependent on PhoP. Overexpression of *cheR* inhibited significantly bacterial intracellular replication, while the *cheA*/*phoP* double deletion mutant failed to replicate in macrophage cells (Fig. 6C).

## DISCUSSION

### Multiple methylated amino acid residues were critical to regulating PhoP activities.

The transcription factor PhoP is highly conserved in bacteria and essential for bacterial virulence. Previous studies showed that PhoP activities could be regulated by acetylation and phosphorylation (17, 18). In this study, we discovered methylation of E8, D9, E107, E108, and R112 of PhoP by mass spectrometry analysis. Comparative sequence analyses of PhoP from six representative bacterial species reveal that these five methylated residues are highly conserved, suggesting their functional importance. In line with these findings, mutations of E8, D9, E107, E108, or R112 to alanine dramatically decreased bacterial survival/proliferation in macrophages but increased bacterial invasion rate. This paradoxical phenotype can be explained by the opposite effect of PhoP on Salmonella pathogenicity island 1 (SPI-1) and SPI-2 (62, 63).

The crystal structure of PhoP gives us some clues why these residues are crucial for PhoP activity. E8 is located in the N-terminal receiver domain of PhoP, and the corresponding residue E7 in E. coli is involved in salt bridges formation with K101 (42). In terms of charge change, glutamate methylation is similar to dephosphorylation, which can neutralize the negative charge of the substrate amino acid (30). We showed that the E8A variant could not be phosphorylated by PAM *in vitro*. This result suggests that E8 is involved in the step of phosphorylation in PhoP activation cascade.

Arginine methylation also plays an important role in regulating protein activity. Methylation of these residues in protein-protein interaction can impair formation of active dimers. For example, Wang et al. reported that R248 methylation impaired the integrity of malate dehydrogenase 1 dimer interface and the consequent formation of active dimmers (26). The α4-β5-α5 structural motif in the PhoP receiver domain is important for PhoP dimerization and function (45). The crystal structure of PhoP from Mycobacterium tuberculosis shows there are salt bridges as well as hydrogen bonds and water-mediated interactions between the side chain of R131 at the center of the dimer interface (64). Further crystal structure analysis showed the relative distance between R112 and DNA was closer than the other residues. Given that R112 of PhoP from Salmonella corresponds with R131 from M. tuberculosis, methylation of R112 is likely to impair PhoP dimerization. Indeed, we found that PhoP purified from *cheR*-overexpressing cells had weaker dimer formation and lower DNA-binding ability compared to PhoP from the wild-type strain, suggesting that CheR-mediated methylation may play an inhibitory role in PhoP activity and, consequently, Salmonella virulence.

### The interplay between CheR and PhoP is crucial for Salmonella stress adaptation.

Bacterial protein methylation is a widespread PTM and is required for virulence in some pathogenic bacteria (31–33). Why Salmonella has methylation on these highly conserved and critical residues of PhoP is intriguing. We raised specific antibodies to synthesized peptides spanning the methylated residues to study their biological functions. Due to the hydrophobicity of neighboring amino acid residues, we only succeeded in generating two specific antibodies against methylated E8 and monomethylated R112. By using these two antibodies combined with pan-methylation antibody, we could monitor the status of PhoP methylation *in vivo*. In-depth studies focusing on E8 and R112 of PhoP showed that the methylation levels of both residues decreased under PhoP/PhoQ-activating conditions and that a low methylation state is beneficial for bacteria to overcome stresses such as low Mg^2+^ and low pH. Consistent with the notion that methylation negatively regulates PhoP activation, overexpression of *cheR* impairs PhoP dimerization and DNA binding.

Methylation is not just a means to regulate PhoP activity. Instead, it could also regulate PhoP stability. Overexpression of *cheR* resulted in the accumulation of PhoP but did not change the transcriptional activity of PhoP. Methylation could protect PhoP from degradation but keep it in an inactive form. Therefore, a pool of hypermethylated, inactive PhoP could accumulate during early infection, allowing initial invasion. A subsequent switch to a state in which hypomethylated PhoP dominates would allow SPI-2 to be activated and favor intracellular bacterial replication. Recalling our previous results showing that acetylation of HilD, a dominant regulator of Salmonella SPI-1, could regulate both its stability and DNA-binding ability to mediate Salmonella virulence (15, 65), we have good reason to believe that PTMs provide an efficient way for bacteria to make the most of proteins.

Colgan et al. showed that lack of *phoP* caused a 10-fold increase of *cheR* transcription in Salmonella (66). Similarly, we found that conditions leading to lower activity of PhoP (e.g., high magnesium concentration) also promoted *cheR* transcription. Although the transcriptional regulation of *cheR* remains unclear in Salmonella, all available data support that the level of *cheR* transcription is negatively correlated with PhoP activity. Therefore, we postulate that the interplay between CheR and PhoP is crucial for Salmonella to deal with stresses, specifically to coordinate chemotaxis and virulence.

### CheR coordinates chemotaxis and virulence.

It is well-known that chemotaxis, or movement under the chemical influence, helps bacteria find optimum environments for their growth and survival. It has been shown that the absence of CheR increased *S.* Typhimurium invasiveness for cultured mammalian cells (67). However, Olsen et al. showed that intestinal invasion of *S.* Typhimurium in the avian host did not depend on chemotaxis, and chemotaxis played a minor role in extra-animal survival of *S.* Typhimurium and Salmonella enterica serovar Dublin (68, 69). Our results showed that overexpression of *cheR* could increase the wild-type *S.* Typhimurium invasion of epithelial cells but not increase the invasion of *phoP* deletion mutant. The discrepancy between these results may be due to the different levels of CheR. Moreover, after being engulfed, Salmonella needs PhoP to activate SPI-2 genes for its survival/replication. In line with this, the transcription of *cheR* decreased dramatically under SPI-2-inducing conditions in *S.* Typhimurium (70). These findings suggest that the expression level of *cheR* must be precisely controlled, and CheR might mediate bacterial invasion through methylating PhoP.

**FIG 7:**
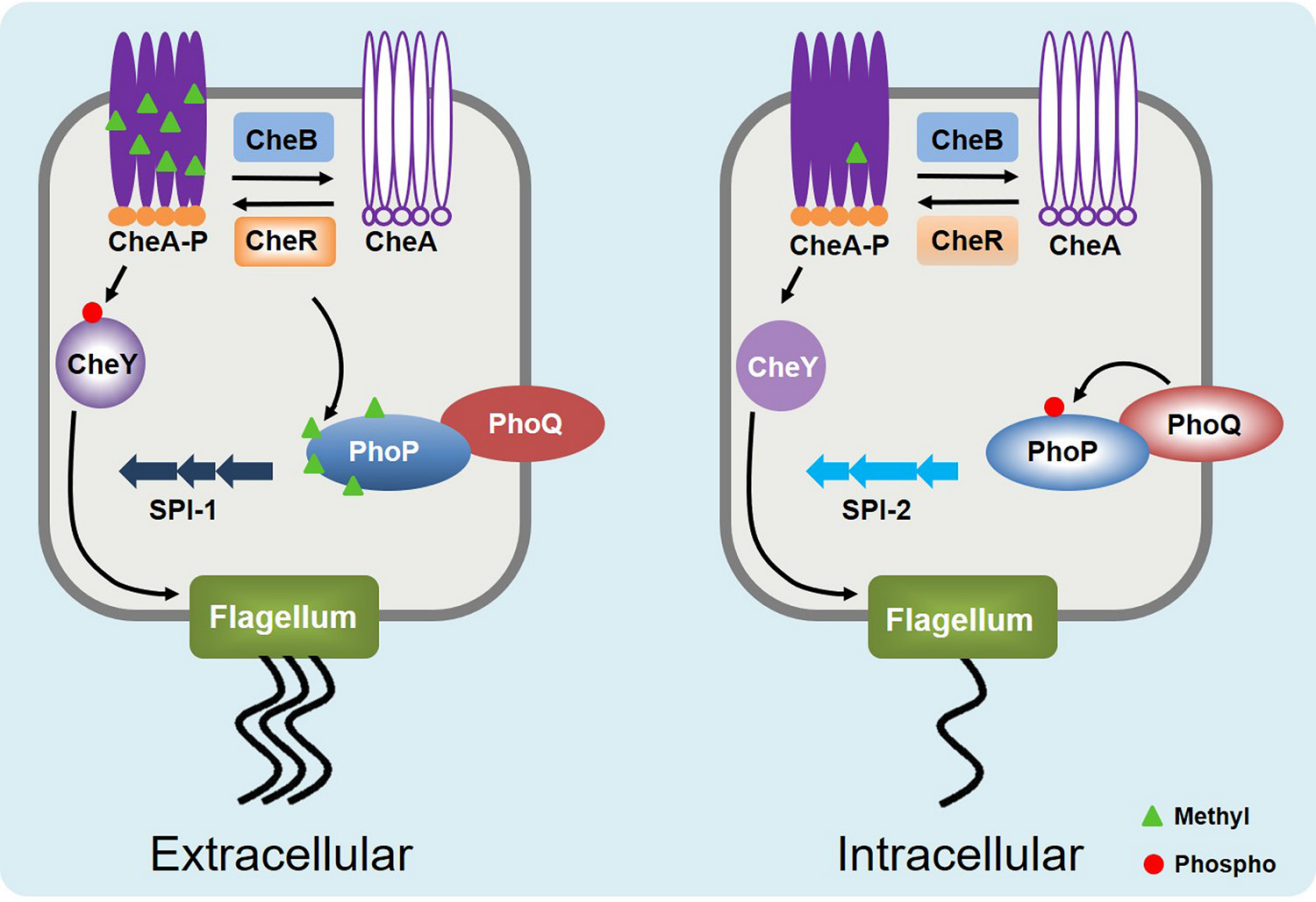
Working model of CheR-mediated methylation in Salmonella invasion and intracellular survival/replication. Salmonella utilizes its chemotaxis system to migrate to epithelial cell surface. Then, CheR inhibits PhoP by methylation, and activated SPI-1 genes promote bacterial invasion. At intracellular stage, the low expression of CheR decreases PhoP methylation level, and activated PhoP facilitates bacterial survival/replication by regulating the transcription of SPI-2 genes.

Therefore, we propose a model to decipher the role of PhoP methylation in Salmonella invasion and intracellular survival/replication (Fig. 7). Initially, Salmonella utilizes the chemotaxis system to migrate to the epithelial cell surface. During this period, PhoP, with a low level of phosphorylation and a high level of methylation, activates the transcription of SPI-1 genes and, consequently, promotes bacterial invasion. After entering the cells, the expression of CheR is downregulated, and hypomethylated PhoP is activated by phosphorylation and then drives the transcription of SPI-2 genes to facilitate bacterial survival/replication. Hypermethylation of PhoP (low phosphorylation) is beneficial during early invasion, while hypomethylated PhoP (high phosphorylation) promotes bacterial intracellular survival/proliferation. Besides phagocytosis of macrophage, the expression of *cheR* was also responsive to PhoP-regulating environmental stimuli (e.g., low Mg^2+^), which indicates that the coordinated expression of PTM enzymes and target protein provides an efficient way for bacteria to adapt to the environment.

### CheR can catalyze methylation on both glutamate and arginine residues.

Protein methylation is known to be one of the most important and common PTMs regulating protein activities (19). To date, there are currently more than 200 known or putative methyltransferases, including lysine methyltransferases and arginine methyltransferases in the human genome (71). Studies of protein methylation have focused heavily on histones or nonhistone proteins from eukaryotes. In contrast, understanding of the biological roles of protein methylation in prokaryotes is extremely limited. Since Ambler et al. first reported the presence of ε-*N*-methyl-lysine in a natural protein in flagellin from *S.* Typhimurium over 50 years ago (72), only a few studies have performed comprehensive analyses of new methylated proteins and their corresponding methyltransferases in bacteria (73–76). Even so, our knowledge of the roles of protein methylation in bacterial virulence remains largely unexplored.

Generally, methyltransferases from eukaryotes act on specific substrate amino acid residues (77). CheR, as a protein methyltransferase, is a key member of the chemotaxis signaling system in bacteria (35). Sensing favorable and deleterious chemicals involves methyl-accepting chemotaxis proteins (MCPs) (78). Methylation is catalyzed by CheR, which reversibly methylates MCPs to form gamma-glutamyl methyl ester residues (78, 79). Conventionally, CheR acts as a glutamate methyltransferase during bacterial chemotaxis, but we provide solid evidence that it also can methylate arginine residue in PhoP. A slight methylation signal was still detected when R112A was incubated with CheR in the presence of ^3^H-SAM, indicating that other residues (e.g., glutamate) can be methylated by CheR. Collectively, CheR can methylate arginine residue as well as glutamate residues in bacteria.

### Multiple methyltransferases are involved in the methylation of PhoP.

The identification of methyltransferase(s) of PhoP is challenging. The dilemma is caused by the intervening of multiple methylation sites and enzymes. As revealed by the autography of *in vitro* methyltransferase assay, CheR and YfcB are the 2 most promising enzymes targeting PhoP. E8 and R112 of PhoP had methylation modifications by Western blotting. Overexpression of *cheR* and *yfcB* could elevate the methylation level of R112, but not E8. Purified CheR, but not YfcB, could effectively catalyze the *in vitro* methylation of R112. PhoP purified from the *cheR*/*yfcB* double mutant strain still had methylation at R112, though much weaker, while the methylation signal at E8 is barely changed. What we conclude is (i) CheR can methylate PhoP, and R112 is its substrate, though we do not exclude the possibility that other residue(s) may be methylated by CheR as well; (ii) another enzyme(s) is also catalyzing the methylation of R112; (iii) YfcB is shown to be a ribosomal protein L3 glutamine methyltransferase (80). The failure to detect glutamine methylation in PhoP by mass spectrometry suggests that there may exist broad substrates of YfcB; and (iv) other unknown methyltransferase(s) might be responsible for the methylation of E8. We acknowledge that overexpression may cause artificial effects. However, the fact that it is hard to observe phenotypes in the deletion mutant of a single methyltransferase highly suggests that multiple methyltransferases may be involved in the methylation of PhoP and functional redundancy may exist. Since bacteria only possess a limited number of protein methyltransferases, we speculated that bacterial protein methyltransferases have to target multiple residues rather than one specific residue. In this sense, the bacterial methylation system is more complicated than we thought and under precise control, which enables bacteria to effectively respond to various environmental stresses with a limited repertoire of methyltransferases.

### PhoP is regulated by multiple PTMs.

Phosphorylation of PhoP is a prominent PTM, which is essential for activating PhoP. Acetylation of K102 or K201 plays an important role in regulating PhoP phosphorylation and DNA-binding activity, respectively (17, 18). Here, we show that another PTM, methylation, is also crucial for the regulation of PhoP activities. Thus, PhoP is regulated by a complex network of PTMs, which may play different roles under different conditions. So far, our findings suggest that both methylation and acetylation inhibit PhoP activities, and these inhibitory strategies are utilized to turn down PhoP activity in favorable environments, while these modifications would be removed in stressful environments. Therefore, PTMs, including methylation, acetylation, and phosphorylation, would work coordinately to enable Salmonella to adapt to various environmental stresses. We believe that multiple PTM-mediated regulation of protein activity might be a universal phenomenon across a wide range of bacterial species, although the coordination mechanism remains unknown.

## MATERIALS AND METHODS

### Strains and media.

Bacterial strains used in this study are listed in Table S1 in the supplemental material. *S.* Typhimurium strain 14028S was used as the wild-type *Salmonella* strain. All mutants derived from strain 14028S were constructed by λ Red recombinase system (81). All constructs were verified by PCR and sequencing. PCR primers are listed in Table S2.

10.1128/mBio.02099-21.7 TABLE S1 Bacterial strains and plasmids used in this study. Download Table S1, DOCX file, 0.02 MB. Copyright © 2021 Su et al. 2021 Su et al. https://creativecommons.org/licenses/by/4.0/ This content is distributed under the terms of the Creative Commons Attribution 4.0 International license.

10.1128/mBio.02099-21.8 TABLE S2 PCR primers used in this study. Download Table S2, DOCX file, 0.03 MB. Copyright © 2021 Su et al. 2021 Su et al. https://creativecommons.org/licenses/by/4.0/ This content is distributed under the terms of the Creative Commons Attribution 4.0 International license.

*S.* Typhimurium strains were grown in lysogeny broth (LB) or M9CA medium with low (8 μM) or high concentration (10 to 50 mM) of Mg^2+^. The final working concentration in media for bacterial selection antibiotic was 100 μg/ml of ampicillin, 50 μg/ml of spectinomycin, or 17 μg/ml of chloramphenicol.

### Plasmid construction.

For overexpression of genes in *S.* Typhimurium, *phoP* and methyltransferase candidate genes were PCR amplified from the genomic DNA and cloned into the *Afe*I and *Fse*I sites of the expression plasmid pQE80 with 6×His tag inserted at the C termini.

### Antibodies.

The following antibodies were used: anti-His peptide monoclonal antibody (Tiangen), anti-Flag peptide monoclonal antibody (Sigma), anti-asymmetric dimethyl arginine motif (ADMA) monoclonal antibody (Cell Signaling), anti-RpoA monoclonal antibody (Santa Cruz), and anti-DnaK antibody (Abcam). Anti-PhoP polyclonal antibody was prepared as follows: the 6×His-tagged PhoP purified from E. coli strain BL21 was used as the antigen to immunize New Zealand rabbits three times to raise polyclonal antibody. For anti-PhoP E8me (α-PhoP E8me) and anti-PhoP R112 monomethylation (α-PhoP R112me1) antibody, the immune peptides VLVVE(Me)DNALLR and IEEVMAR(Me)MQALMRR were used as antigen to immunize rabbits, respectively. Nonspecific antibodies were removed by incubating with control peptide column (VLVVEDNALLR or IEEVMARMQALMRR, respectively).

### Purification of proteins.

PhoP, PhoP variant proteins, and potential PhoP methyltransferases were purified as previously described (17). Briefly, the constructed plasmids were transformed individually into E. coli strain BL21, and the resultant strains were grown in LB medium with 100 μg/ml of ampicillin added by aerobic shaking at 37°C. IPTG (isopropyl-β-d-thiogalactopyranoside) was added to a final concentration of 0.1 mM when the absorbance of the culture reached an optical density at 600 nm (OD_600_) of ∼0.6. Incubation was continued for 5 h at 30°C. Cells were harvested and lysed in lysis buffer with 50 mM Tris-HCl (pH 7.5), 0.6 M NaCl, 10% (vol/vol) glycerol, and 5 mM phenylmethylsulfonyl fluoride (PMSF) by high-pressure homogenization. The supernatant obtained after centrifugation (15,000 × *g* for 30 min at 4°C) was loaded on an Ni-NTA column (GE Healthcare, USA) preequilibrated with lysis buffer. The column was subsequently washed with 20 ml of binding buffer (20 mM Tris-HCl, 0.5 M NaCl, 10% [vol/vol] glycerol, pH 7.6) plus 20 mM imidazole and 40 mM imidazole, respectively, and the histidine-tagged protein was eluted with 2 ml of binding buffer containing 300 mM imidazole. Purity was assessed by SDS-PAGE.

### Identification of methylation by mass spectrometry.

The wild-type *S.* Typhimurium cells were grown in LB broth to log or stationary phase or in M9CA medium with low or high concentration of Mg^2+^ supplemented until an OD_600_ of ∼1.0 was reached. PhoP proteins were purified from the above four conditions and separated on 12% SDS-PAGE. The excised bands were destained and dehydrated. In order to digest the samples by trypsin, the proteins were pretreated with 10 mM dithiothreitol (DTT) in 100 mM NH_4_HCO_3_ at 56°C for 30 min and then incubated with 55 mM iodoacetamide (IAM) in 100 mM NH_4_HCO_3_ at room temperature for 20 min (in complete darkness). Afterwards, the protein samples were digested in-gel with trypsin at 37°C for another 20 h. Peptides were separated by the Easy-nLC high-performance liquid chromatography (HPLC) system (Thermo Fisher Scientific) and analyzed by Q-Exactive mass spectrometry (Thermo Fisher Scientific). Mass spectrometric data were analyzed using the Mascot 2.2 software for database search. To quantify the methylation levels of R112, the ratios of average area (representing peptide intensity) of R112-methylated peptides to the average area of total R112-containing peptides were calculated.

### Quantitative real-time PCR assay.

Total RNA was isolated from the wild-type *S.* Typhimurium and *phoP* mutant cells using TRIzol (Thermo Fisher Scientific). Contaminated genomic DNA in RNA samples was removed by treatment with RNase-free DNase (Thermo Fisher Scientific). RNA samples were reversed transcribed with the random hexamers by using SuperScript III first-strand synthesis system (Thermo Fisher Scientific). Comparative quantitative real-time PCR (qPCR) analysis was performed using SYBR Premix Ex Taq II (TaKaRa) in the QuantStudio3 fast real-time PCR system (Thermo Fisher Scientific) with each primer set (Table S2). All reactions were performed in triplicate. Fold changes of gene expression in *phoP* mutant strains compared to the wild type were calculated by using the threshold cycle (2^−ΔΔ^*^CT^*) method. We used 16S rRNA as an internal control.

### Bacterial spot plating assay.

Bacterial sensitivity to a low concentration (8 μM) of Mg^2+^ was evaluated by a previously described method with some modifications (82). *S.* Typhimurium cells were grown at 37°C in liquid LB medium overnight and were then normalized to OD_600_ of 0.1 and continued to grow in liquid M9CA medium supplemented with 8 μM Mg^2+^. Samples were collected at the indicated time points (in hours), normalized to the same OD_600_, serially 5-fold diluted, and spotted (2 μl of dilutions) on LB agar plates. Plates were photographed after 14 h of incubation at 37°C.

### Log-phase ATR.

Log-phase ATR was measured as described previously (17). Briefly, cells were grown overnight in Minimal E glucose (EG) medium (MgSO4, 0.098 g/l; citric acid monohydrate, 2.0 g/l; K_2_HPO_4_ ·3H_2_O, 13.1 g/l; NaNH_4_HPO_4_, 2.29 g/l; glucose, 0.4% [wt/vol], pH 7.7) (the pH of culture medium was adjusted with HCl), and then the overnight culture was diluted 1:100 in 5.0 ml of fresh EG medium at pH 7.7. When the cultures reached OD_600_ of 0.4, cells were prepared by acid shock treatment in EG medium at pH 5.8 for 1 h before shift to pH 3.3. Cell viability was measured by counting colonies generated by viable adapted cells at 0 h, 1 h, and 2 h post-acid challenge. The experiment was repeated independently three times, and a representative result was shown.

### Phos-tag acrylamide gel analysis.

PhoP phosphorylation by phosphoramidate (PAM; ≥90% purity; custom-made) was investigated by using Phos-tag SDS-PAGE followed by Western blotting. Purified PhoP was added to phosphorylation buffer (50 mM HEPES [pH 7.5], 100 mM NaCl, and 10 mM MgCl_2_) supplemented with 20 mM PAM, and the mixtures were incubated at 37°C for 1 h. The reactions were stopped by adding sample loading buffer. Phos tag gels were prepared according to the instructions described by the manufacturer with minor modifications. Phos tag acrylamide running gel contained 10% (wt/vol) 29:1 acrylamide/*N*,*N*′-methylene-bis-acrylamide, 375 mM Tris (pH 8.8), and 0.1% (wt/vol) SDS. Gels were copolymerized with 50 μM Phos tag acrylamide and 100 μM MnCl_2_ for analysis of the PAM-treated proteins. All the Phos tag gels were run at 4°C at 30 mA constant current. After the electrophoresis was complete, the proteins were transferred to polyvinylidene difluoride (PVDF) membranes and detected by Western blotting.

### Protein cross-linking.

Purified PhoP was incubated with 1 mM dextran sulfate sodium (DSS) in 50 mM HEPES-Na (pH 7.5), 150 mM NaCl, 10 mM MgCl_2_, 20% glycerol, and 0.05% Tween 20 at 25°C for 30 min (83). The reaction was terminated by the addition of sample loading buffer, and then samples were analyzed by Western blotting.

### Electrophoretic mobility shift assay.

Electrophoretic mobility shift assays (EMSAs) were performed using the purified PhoP or its variants and a 184-bp promoter sequence of *phoP* (17). DNA probes were PCR amplified using primers listed in Table S2. The probe (1.6 pmol) was mixed with various amounts of proteins in 10 μl of EMSA binding buffer (25 mM Tris-HCl, 50 mM KCl, 5 mM MgCl_2_, 0.5 mM EDTA, and 10% glycerol, pH 8.0). After incubation at room temperature for 20 min, the samples were analyzed by 5% polyacrylamide gel electrophoresis (90 V for 45 min for sample separation). The gels were subjected to DNA dye for 5 min and photographed by using a gel imaging system (Protein Simple). The assay was repeated at least three times, and a representative result was shown.

### Isothermal titration calorimetry.

Isothermal titration calorimetry (ITC) was performed using a MicroCal PEAQ-ITC instrument (Malvern). PhoP box consensus sequence DNA at a concentration of 6.15 μM was titrated into a cell containing 0.6 μM PhoP or methylated PhoP at 25°C with a stirring speed of 1,000 rpm. Every injection volume for the DNA was 1.96 μl. Consensus sequence DNA was resuspended in buffer to match the desalting buffer. Data fitting was performed with the NanoAnalyze software (Malvern).

### Protein concentration.

Protein concentration was determined using the Bradford reagent with bovine serum albumin (BSA) as a standard (84).

### Immunoprecipitation.

Mouse macrophage-like RAW 264.7 cells were seeded in 10-cm dishes with 1.5 × 10^7^ cells per dish and infected with bacteria at a multiplicity of infection (MOI) of 10 for 1 h. Then, cells were treated with 100 μg/ml gentamicin for 2 h to kill extracellular bacteria, and cells were continued in culture with media containing 25 μg/ml gentamicin for 24 h. Infected cells were lysed with 0.025% (vol/vol) SDS in PBS after washing three times with PBS. Intracellular bacteria were harvested for immunoprecipitation, while bacteria cultured in Dulbecco’s modified Eagle medium (DMEM) were used as control. Flag-tagged PhoP was immunoprecipitated according to Crosslink immunoprecipitation (IP) kit protocol (Thermo Fisher Scientific).

### Chloramphenicol half-life assay.

The half-life of PhoP was determined as previously described (15). Briefly, the empty vector or *cheR*-expressing plasmid was transformed into bacteria. IPTG was used to induce the expression of CheR for 1 h in M9CA medium with a low concentration of Mg^2+^. The cells were treated with 1 mg/ml of chloramphenicol to block the translation and then harvested at indicated time points for Western blot analysis.

### *In vitro* methyltransferase assay.

We incubated 1.4 μg of purified recombinant PhoP proteins at 30°C overnight with 2.8 μg of recombinant putative protein methyltransferases in 20 μl of methylation buffer (20 mM Tris-HCl, 150 mM NaCl, and 1 mM EDTA, pH 7.5) supplemented with 1 μCi of *S*-adenosyl-l-[*methyl*-^3^H] methionine (PerkinElmer) or 0.2 mM cold SAM (NEB). Reactions were stopped by the addition of 2×SDS-PAGE sample buffer and heating. Samples were separated in 12% SDS-PAGE and followed by Western blotting or autoradiography. For autoradiography, the gels were fully dry by gel dryer (Wadali) and then exposed to X-ray film.

### Cell infection assays.

HeLa cell invasion assay was performed as described previously (15). Briefly, for epithelial cell infection, HeLa cells were seeded at 1 × 10^5^ cells per well in 24-well plates and infected at an MOI of 100 with overnight-cultured *S.* Typhimurium. After 1 h of infection, cells were washed twice with DMEM and incubated in DMEM-10% fetal bovine serum (FBS) plus 100 mg/ml gentamicin for 2 h to kill extracellular bacteria and then were washed twice with DMEM and lysed as described above. Lysates were plated with the appropriate dilution, followed by viable bacteria being counted to calculate the invasion efficiency (percentage of the starting inoculum internalized at the end of the assay). Each invasion assay was performed simultaneously in 2 separate wells and repeated 3 times.

For macrophage infection assay, 2 × 10^5^ mouse macrophage-like RAW 264.7 cells/well were seeded into 24-well plates in triplicates. Cells were infected at an MOI of 10 with overnight-cultured bacteria. After extracellular bacteria were removed by extensive washing with DMEM 1 h after infection, infected cells were incubated in fresh tissue culture medium containing 100 μg/ml gentamicin for the first 2 h postinfection and 25 μg/ml gentamicin for the remainder of the experiment. Infected cells were lysed at the desired postinfection time points with 0.025% (vol/vol) SDS in PBS after washing three times with PBS. The number of viable intracellular bacteria were determined by serial dilutions and plating on LB agar plates. Intracellular bacterial growth was measured as the fold change in CFU per milliliter recovered from macrophages between two time points (85). All experiments were performed in triplicate.

### Animal studies.

Six to 8-week-old female BALB/c mice were used in this study. All mice were housed in sterilized cages under standard room conditions of temperature, humidity, and free access to food and water and were randomly divided into indicated groups (seven mice each group).

For oral infection, mice were derived of food and water for 4 h prior to administration of 20 mg of streptomycin by oral gavage. Afterward, food and water were provided *ad libitum*. At 20 h after streptomycin treatment, food and water were withdrawn for another 4 h, followed by intragastric injection with 1.5 × 10^7^ CFU of bacteria per mouse. Thereafter, water and food were provided *ad libitum* 4 h postinfection. For intraperitoneal injection, each mouse was given 1.5 × 10^5^ CFU of bacteria per mouse. The PBS control group was set up under the same conditions. Mice were observed regularly to record the mortality rate.

To assess the numbers of *S.* Typhimurium in livers and spleens, the specimens were harvested 48 h after oral injection and homogenized in 5 ml of PBS solution. Tenfold serial dilutions of organ homogenates were plated on LB agar plates and incubated for 24 h at 37°С. Afterward, the colony counts in the liver and spleen were determined. The immunohistochemistry was performed as described previously (86). Cecal were fixed in 4% paraformaldehyde overnight to prepare paraffin blocks for hematoxylin and eosin (H&E) staining.

### Ethics statement.

All animal experiments were approved by Shanghai Jiao Tong University School of Medicine, and these tests were performed with strict observance of the National Research Council Guide for Care and Use of Laboratory Animals (SYXK [Shanghai 2007-0025]).
