# Supplementary figures and images for: Methylation of PhoP by CheR Regulates Salmonella Virulence
Source: mBio. 2021 Sep 21;12(5):e02099-21. doi: 10.1128/mBio.02099-21 (PMC8546571; doi:10.1128/mBio.02099-21)

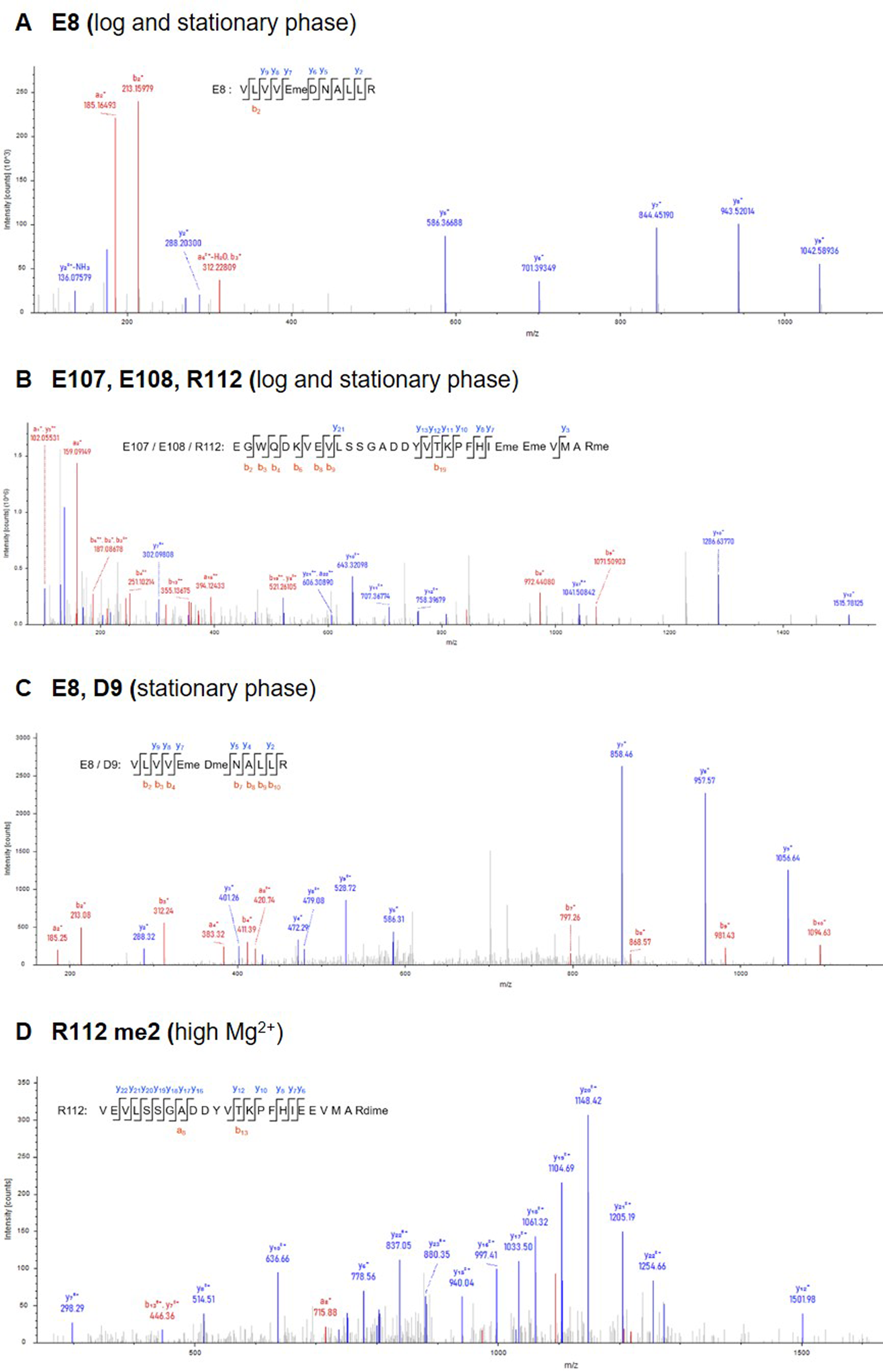

Supplement: FIG S1 [file mbio.02099-21-sf001.tif]

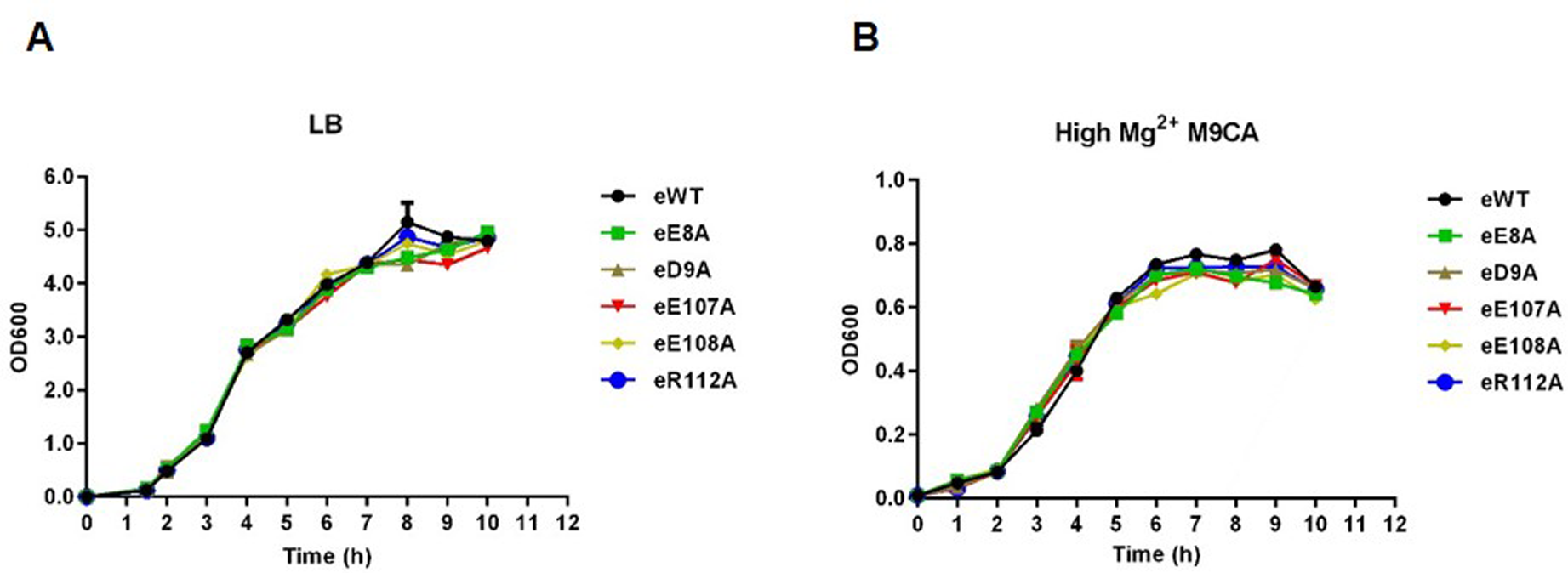

Supplement: FIG S2 [file mbio.02099-21-sf002.tif]

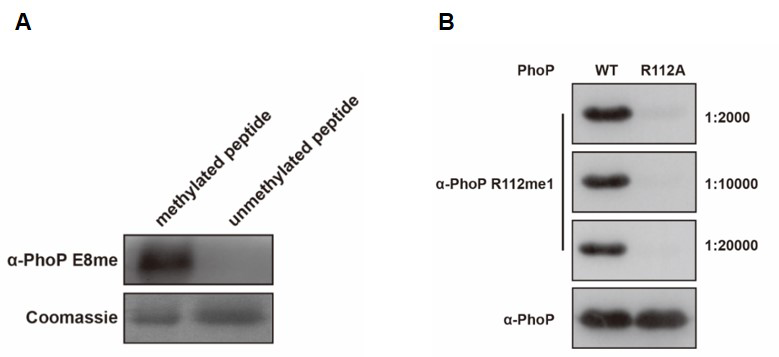

Supplement: FIG S5 [file mbio.02099-21-sf005.tif]

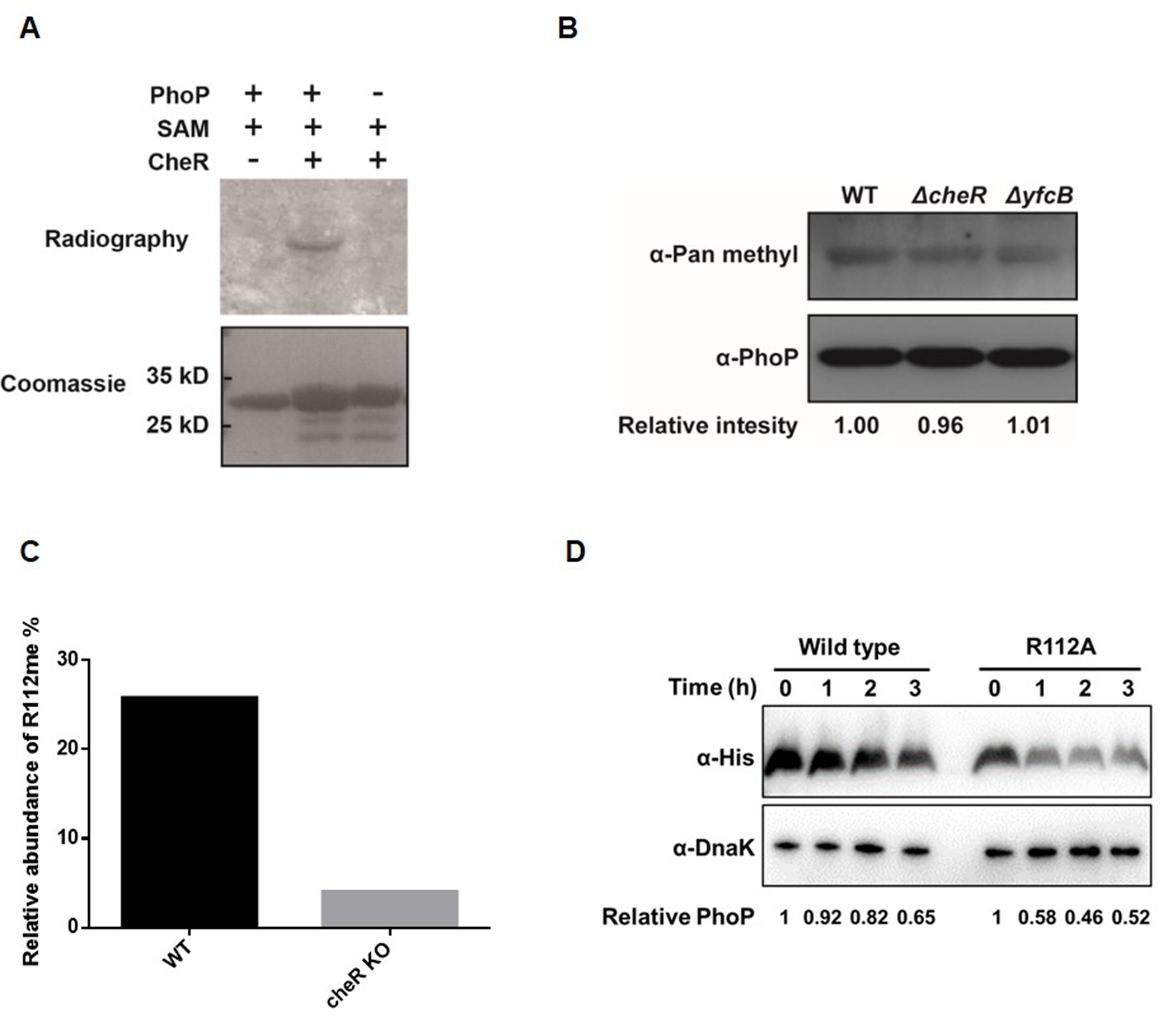

Supplement: FIG S6 [file mbio.02099-21-sf006.tif]
