# Supplementary material for: Methylation of PhoP by CheR Regulates Salmonella Virulence
Source: mBio. 2021 Sep 21;12(5):e02099-21. doi: 10.1128/mBio.02099-21 (PMC8546571; doi:10.1128/mBio.02099-21)
Supplement: TABLE S1 [file mbio.02099-21-st001.docx]

**Table S1.** Bacterial strains and plasmids used in this study

| **Strains and plasmids** | **Genotypes and characteristics** | **Sources or references** |
| --- | --- | --- |
| **Strains** |  |  |
| *E. coli* DH5α | F- 80*lacZ* M15 (lacZYA–argF)U169*eoR recA1 endA1 hsdR17 phoA supE*44-thi-1 *gyrA96 relA1* | Laboratory stock |
| *E. coli* BL21 | F- *ompT gal dcm lon hsdSB*(rB- mB-) λ(DE3 [*lacI lacUV*5-T7 gene 1 *ind1 sam7 nin5*]) | Laboratory stock |
| *S.* Typhimurium14028S | Wild type *S.* Typhimurium strain | Laboratory stock |
| Δ*phoP* | *S.* Typhimurium 14028S Δ*phoP* | Laboratory stock |
| *phoP*-Flag | *S.* Typhimurium 14028S *phoP*-Flag chromosome mutation | Laboratory stock |
| *phoP* (E8A)-Flag | *S.* Typhimurium 14028S *phoP* (E8A)-Flag chromosome mutation | This study |
| *phoP* (D9A)-Flag | *S.* Typhimurium 14028S *phoP* (D9A)-Flag chromosome mutation | This study |
| *phoP* (E107A)-Flag | *S.* Typhimurium 14028S *phoP* (E107A)-Flag chromosome mutation | This study |
| *phoP* (E108A)-Flag | *S.* Typhimurium 14028S *phoP* (E108A)-Flag chromosome mutation | This study |
| *phoP* (R112A)-Flag | *S.* Typhimurium 14028S *phoP* (R112A)-Flag chromosome mutation | This study |
| *phoP* (eWT)-His | *S.* Typhimurium 14028S *phoP*-His chromosome strain | Laboratory stock |
| *phoP* (eE8A)-His | *S.* Typhimurium 14028S *phoP* (E8A)-His chromosome mutation | This study |
| *phoP* (eD9A)-His | *S.* Typhimurium 14028S *phoP* (D9A)-His chromosome mutation | This study |
| *phoP* (eE107A)-His | *S.* Typhimurium 14028S *phoP* (E107A)-His chromosome mutation | This study |
| *phoP* (eE108A)-His | *S.* Typhimurium 14028S *phoP* (E108A)-His chromosome mutation | This study |
| *phoP* (eR112A)-His | *S.* Typhimurium 14028S *phoP* (R112A)-His chromosome mutation | This study |
| Δ*yfcB* | *S.* Typhimurium 14028S Δ*yfcB* | This study |
| Δ*yafE* | *S.* Typhimurium 14028S Δ*yafE* | This study |
| Δ*yafS* | *S.* Typhimurium 14028S Δ*yafS* | This study |
| Δ*tehB* | *S.* Typhimurium 14028S Δ*tehB* | This study |
| ΔSTM14_1982 | *S.* Typhimurium 14028S ΔSTM14_1982 | This study |
| Δ*yecO* | *S.* Typhimurium 14028S Δ*yecO* | This study |
| Δ*cheR* | *S.* Typhimurium 14028S Δ*cheR* | This study |
| Δ*ubiG* | *S.* Typhimurium 14028S Δ*ubiG* | This study |
| Δ*yjhP* | *S.* Typhimurium 14028S Δ*yjhP* | This study |
| Δ*cheA* | *S.* Typhimurium 14028S Δ*cheA* | This study |
| Δ*cheA*/*phoP* | *S.* Typhimurium 14028S Δ*cheA*/*phoP* | This study |
|  |  |  |
| Δ*cheR*/*yfcB* | *S.* Typhimurium 14028S Δ*cheR*/*yfcB* | This study |
| **Plasmids** |  |  |
| pKD46 | Express λ red recombinase | Laboratory stock |
| pKD3 | Source for chloramphenicol resistance cassette (cat) | Laboratory stock |
| pCP20 | FLP recombinase | Laboratory stock |
| pQE80 | Expression vector | Laboratory stock |
| pQE80-*yafE* | Amp^R^, pQE80 harboring *yafE* | This study |
| pQE80-*yafS* | Amp^R^, pQE80 harboring *yafS* | This study |
| pQE80-*tehB* | Amp^R^, pQE80 harboring *tehB* | This study |
| pQE80-STM14_1982 | Amp^R^, pQE80 harboring STM14_1982 | This study |
| pQE80-*yecO* | Amp^R^, pQE80 harboring *yecO* | This study |
| pQE80-*cheR* | Amp^R^, pQE80 harboring *yafS* | This study |
| pQE80-*ubiG* | Amp^R^, pQE80 harboring *yafE* | This study |
| pQE80-*yjhP* | Amp^R^, pQE80 harboring *yjhP* | This study |
| pQE80-*yfcB* | Amp^R^, pQE80 harboring *yfcB* | This study |
| pQE80-*phoP* | Amp^R^, pQE80 harboring *phoP* | This study |
| pQE80-*phoP* (E8A) | Amp^R^, pQE80 harboring *phoP* (E8A) | This study |
| pQE80-*phoP* (D9A) | Amp^R^, pQE80 harboring *phoP* (D9A) | This study |
| pQE80-*phoP* (E107A) | Amp^R^, pQE80 harboring *phoP* (E107A) | This study |
| pQE80-*phoP* (E108A) | Amp^R^, pQE80 harboring *phoP* (E108A) | This study |
| pQE80-*phoP* (R112A) | AmpR, pQE80 harboring *phoP* (R112A) | This study |
