# Supplementary material for: Methylation of PhoP by CheR Regulates Salmonella Virulence
Source: mBio. 2021 Sep 21;12(5):e02099-21. doi: 10.1128/mBio.02099-21 (PMC8546571; doi:10.1128/mBio.02099-21)
Supplement: TABLE S2 [file mbio.02099-21-st002.docx]

**Table S2.** PCR primers used in this study

| **Primer Name** | **Primer Sequence** |
| --- | --- |
| *phoP*-qPCR-F | tgcgcgtactggttgtagag |
| *phoP*-qPCR-R | tcatccggcagacctaaatc |
| *pmrA*-qPCR-F | cataataaccagggtgaaagtgaac |
| *pmrA*-qPCR-R | cgttatcccagttgtagatatcgtt |
| *mgtA*-qPCR-F | aatcctttcaacatcttactcacga |
| *mgtA*-qPCR-R | attttcattaataacccgcagtacg |
| *mgtC*-qPCR-F | agggagaaaaacgttatatcctgaa |
| *mgtC*-qPCR-R | atttctttatagccctgttcctgag |
| *pagC*-qPCR-F | acatttaaagaacattccactcagg |
| *pagC*-qPCR-R | agccgtttatttttgtagaggagat |
| *cheR*-qPCR-F | tacagatgacacagcgcctc |
| *cheR*-qPCR-R | atcatccagtcccagggcac |
| *sipC*-qPCR-F | gtaccgatgcgacgaaaaat |
| *sipC*-qPCR-R | atcgattcgggtcatatcca |
| *sseL*-qPCR-F | agttcgctcagacagatcaag |
| *sseL*-qPCR-R | aggatgaatcagcccaatagg |
| *ssaV*-qPCR-F | ggtatcgagagggtggcgga |
| *ssaV* -qPCR-R | ccgtccatcgcaccgagaaa |
| *spiC*-qPCR-F | gggcatcctgccagaggaga |
| *spiC*-qPCR-R | catcccccatccgctgtgag |
| *sifA*-qPCR-F | ttgcgatgcgcaggctaact |
| *sifA*-qPCR-R | gcaaagcaaaagcggaccgt |
| genome-*phoP* mutant-F1 | ctgattactaccttaatgaa |
| genome-*phoP* mutant-R1 | gaagcagctccagcctacacttagcgcaattcaaaaagat |
| genome-*phoP* mutant-F2 | atctttttgaattgcgctaagtgtaggctggagctgcttc |
| genome-*phoP* mutant-R2 | tggcgagcaaatttattcatcatatgaatatcctccttag |
| genome-*phoP* mutant-F3 | ctaaggaggatattcatatgatgaataaatttgctcgcca |
| genome-*phoP* mutant-R3 | tcacgtacttctttgagttt |
| genome-*phoP* mutant-check-F | caccacctgaaggttcagct |
| genome-*phoP* mutant-check-R | cgccgtggcaggataaatat |
| pQE80-*phoP*-F | atgatgcgcgtactggttgt |
| pQE80-*phoP*-R | tataggccggcctgcgcaattcaaaaagatatc |
| pQE80-*yafE*-F | atgacaacgcattcccaccat |
| pQE80-*yafE*-R | tataggccggccttaccgctttatgcgcttc |
| pQE80-*yafS*-F | atgaaaccggcaaggctctc |
| pQE80-*yafS*-R | tataggccggcctgccatccggtttgcgat |
| pQE80-*tehB*-F | atgaccgttcgtgacgaaaattac |
| pQE80-*tehB*-R | tataggccggcctggccgtttttctcgccag |
| pQE80-STM14_1982-F | atggatttaaatccggatagcc |
| pQE80-STM14_1982-R | tataggccggcctcgatgagcgtggcgtag |
| pQE80-*yecO*-F | atgtctcaccgcgacacgctt |
| pQE80-*yecO*-R | tataggccggccttgcggcaactccggcttt |
| pQE80-*cheR*-F | atgacatcatctctgccctc |
| pQE80-*cheR*-R | tataggccggccttgctttatccttacttagc |
| pQE80-*ubiG*-F | atgaacactgaaaaaccgtcg |
| pQE80-*ubiG*-R | tataggccggcctggcttttttagcgcgggt |
| pQE80-*yjhP*-F | atgatgatggatatcagcgcc |
| pQE80-*yjhP*-R | tataggccggccttcgcgcgattaacgcaaatacg |
| pQE80-*yfcB*-F | gtggataaaattttcgtcgatg |
| pQE80-*yfcB*-R1 | tataggccggccttaatctttataaatattgaaatgttcacgg |
| pQE80-*yfcB*-R2 | tataggccggcctatctttataaatattgaaatgttcacgg |
| genome-*yfcB* mutant-F1 | cggcgtttgctgtttcagaat |
| genome-*yfcB* mutant-R1 | gaagcagctccagcctacacgtattcctccagggcatgct |
| genome-*yfcB* mutant-F2 | agcatgccctggaggaatacgtgtaggctggagctgcttc |
| genome-*yfcB* mutant-R2 | cgttgttgtcgtttgcgtgttcatatgaatatcctcctta |
| genome-*yfcB* mutant-F3 | taaggaggatattcatatgaacacgcaaacgacaacaacg |
| genome-*yfcB* mutant-R3 | gcatcgggacaaaagaacgg |
| genome-*yfcB* mutant-check-F | ccgtgattttcttgcgcagt |
| genome-*yfcB* mutant-check-R | cacctcgcgctatactaccc |
| *yafE*-KO-F | tagccgccgaatttaatgcgagcatgccctggaggaatacgtgtaggctggagctgcttc |
| *yafE*-KO-R | gccatcacggctccgttatcgttgttgtcgtttgcgtgttcatatgaatatcctccttagttcc |
| *yafE*-KO-check-F | ctgtcttcgtggcaaactag |
| *yafE*-KO-check-R | tcgagatcgtgttgcagatc |
| *yafS*-KO-F | tactgttaagattcatatattacctctcaaataattagcggaaggttgcggtgtaggctggagctgcttc |
| *yafS*-KO-R | gccgcggcaatgaatcccacgcaggaagatagtggctaccaggcggaagcggtccatatgaatatcctcctta |
| *yafS*-KO-check-F | cctccggcatccacttatgtt |
| *yafS*-KO-check-R | gtctcgatgctgcgttaggt |
| genome-*tehB* mutant-F1 | acacgctggcgaaaatgctat |
| genome-*tehB* mutant-R1 | gaagcagctccagcctacacttttttcctccgttttcaacagtg |
| genome-*tehB* mutant-F2 | cactgttgaaaacggaggaaaaaagtgtaggctggagctgcttc |
| genome-*tehB* mutant-R2 | acttacagaacttgcatcgccatatgaatatcctccttagttcc |
| genome-*tehB* mutant-F3 | ggaactaaggaggatattcatatggcgatgcaagttctgtaagt |
| genome-*tehB* mutant-R3 | gaaatgtggatctacccagc |
| genome-*tehB* mutant-check-F | ctggctgagcgtcaatggc |
| genome-*tehB* mutant-check-R | gacgggatagtatgttatcgg |
| STM14_1982-KO-F | gtacaggttattacctgaaacatgcacctgcgactcatgccatttcgctggtgtaggctggagctgcttc |
| STM14_1982-KO-R | cgggttaaacccacatgatcgcgctgccgaccgctcggaagtgcctgtacggtccatatgaatatcctcctta |
| STM14_1982-KO-check-F | gtgtcctactgatacgcgact |
| STM14_1982-KO-check-R | gtctaaccaggacccgtgac |
| genome-*yecO* mutant-F1 | catggcaacgcggtggaata |
| genome-*yecO* mutant-R1 | gaagcagctccagcctacacaacgtatacatccgggaaaaat |
| genome-*yecO* mutant-F2 | atttttcccggatgtatacgttgtgtaggctggagctgcttc |
| genome-*yecO* mutant-R2 | ctgataaaagttaccaaactcgacatatgaatatcctccttagttcc |
| genome-*yecO* mutant-F3 | ggaactaaggaggatattcatatgtcgagtttggtaacttttatcag |
| genome-*yecO* mutant-R3 | aaagctgcgtaggatcgatg |
| genome-*yecO* mutant-check-F | cggttttagcgaattgcaaagc |
| genome-*yecO* mutant-check-R | gctgatcgttacccaacagt |
| *cheR*-KO-F | gcgcccgttgtactttgaatgtgattaagaaggcgctatgacatcgtgtaggctggagctgcttc |
| *cheR*-KO-R | catcaactgacaataccctgattttactcatgctttatccttacttagcgcatatgaatatcctccttagttcc |
| *cheR*-KO-check-F | attagccgccagggatgatg |
| *cheR*-KO-check-R | agcgaagagaccatcaccac |
| *ubiG*-KO-F | atagatcaatagcggaatctcatgtactcgtgtaggctggagctgcttc |
| *ubiG*-KO-R | cgcaacccttataggaaaattctttgatgcatatgaatatcctcctta |
| *ubiG*-KO-check-F | ctgtaattttatcacgcacc |
| *ubiG*-KO-check-R | gaatccctactagaatactc |
| *yjhP*-KO-F | tacaaccgggctgaacaacgttcgcgcccagcctgacaatcgctaaggaggtgtaggctggagctgcttc |
| *yjhP*-KO-R | tacttttaaatggaggtatacctcttgtaggctatacctcctgcttcacgggtccatatgaatatcctcctta |
| *yjhP*-KO-check-F | accgccgttgagtgctatag |
| *yjhP*-KO-check-R | gggcagtgctcaaaatcctc |
| *phoP* E8A-F | cgcgtactggttgtagcggataatgcattattac |
| *phoP* E8A-R | gtaataatgcattatccgctacaaccagtacgcg |
| *phoP* D9A-F | cgtactggttgtagaggctaatgcattattacgcc |
| *phoP* D9A-R | ggcgtaataatgcattagcctctacaaccagtacg |
| *phoP* E107A-F | gaagccattccacatcgcagaggtaatggcgcg |
| *phoP* E107A-R | cgcgccattacctctgcgatgtggaatggcttc |
| *phoP* E108A-F | ccattccacatcgaagcggtaatggcgcgtatg |
| *phoP* E108A-R | catacgcgccattaccgcttcgatgtggaatgg |
| *phoP* R112A-F | cgaagaggtaatggcggctatgcaggcgttaatg |
| *phoP* R112A-R | cattaacgcctgcatagccgccattacctcttcg |
| P*phoP*_F for EMSA | ctgaaagagttgacccgtgg |
| P*phoP*_R for EMSA | atcctctacaaccagtacgc |
